# Breast cancer growth and proliferation is suppressed by the mitochondrial targeted furazano[3,4-b]pyrazine BAM15

**DOI:** 10.1186/s40170-021-00274-5

**Published:** 2021-10-09

**Authors:** Elizabeth R. M. Zunica, Christopher L. Axelrod, Eunhan Cho, Guillaume Spielmann, Gangarao Davuluri, Stephanie J. Alexopoulos, Martina Beretta, Kyle L. Hoehn, Wagner S. Dantas, Krisztian Stadler, William T. King, Kathryn Pergola, Brian A. Irving, Ingeborg M. Langohr, Shengping Yang, Charles L. Hoppel, L. Anne Gilmore, John P. Kirwan

**Affiliations:** 1 Integrated Physiology and Molecular Medicine Laboratory, Pennington Biomedical Research Center, 6400 Perkins Road, Baton Rouge, LA 70808 USA; 2 Department of Nutrition, Case Western Reserve University, Cleveland, OH 44109 USA; 3 Clinical Oncology and Metabolism, Pennington Biomedical Research Center, Baton Rouge, LA 70808 USA; 4 Department of Translational Services, Pennington Biomedical Research Center, Baton Rouge, LA 70808 USA; 5 School of Kinesiology, Louisiana State University, Baton Rouge, LA USA; 6 Sarcopenia and Malnutrition Laboratory, Pennington Biomedical Research Center, Baton Rouge, LA 70808 USA; 7 School of Biotechnology and Biomolecular Sciences, University of New South Wales, Sydney, New South Wales 2052 Australia; 8 Department of Oxidative Stress and Disease, Pennington Biomedical Research Center, Baton Rouge, LA 70808 USA; 9 Department of Pathobiological Sciences, Louisiana State University, Baton Rouge, LA 70803 USA; 10 Department of Biostatistics, Pennington Biomedical Research Center, Baton Rouge, LA 70808 USA; 11 Department of Pharmacology, Case Western Reserve University, Cleveland, OH 44109 USA; 12 Department of Clinical Nutrition, University of Texas Southwestern Medical Center, Dallas, TX 75390 USA

**Keywords:** BAM15, Breast cancer, Tumor metabolism, Mitochondrial function, Cell proliferation

## Abstract

**Background:**

Enhanced metabolic plasticity and diversification of energy production is a hallmark of highly proliferative breast cancers. This contributes to poor pharmacotherapy efficacy, recurrence, and metastases. We have previously identified a mitochondrial-targeted furazano[3,4-b]pyrazine named BAM15 that selectively reduces bioenergetic coupling efficiency and is orally available. Here, we evaluated the antineoplastic properties of uncoupling oxidative phosphorylation from ATP production in breast cancer using BAM15.

**Methods:**

The anticancer effects of BAM15 were evaluated in human triple-negative MDA-MB-231 and murine luminal B, ERα-negative EO771 cells as well as in an orthotopic allograft model of highly proliferative mammary cancer in mice fed a standard or high fat diet (HFD). Untargeted transcriptomic profiling of MDA-MB-231 cells was conducted after 16-h exposure to BAM15. Additionally, oxidative phosphorylation and electron transfer capacity was determined in permeabilized cells and excised tumor homogenates after treatment with BAM15.

**Results:**

BAM15 increased proton leak and over time, diminished cell proliferation, migration, and ATP production in both MDA-MB-231 and EO771 cells. Additionally, BAM15 decreased mitochondrial membrane potential, while inducing apoptosis and reactive oxygen species accumulation in MDA-MB-231 and EO771 cells. Untargeted transcriptomic profiling of MDA-MB-231 cells further revealed inhibition of signatures associated with cell survival and energy production by BAM15. In lean mice, BAM15 lowered body weight independent of food intake and slowed tumor progression compared to vehicle-treated controls. In HFD mice, BAM15 reduced tumor growth relative to vehicle and calorie-restricted weight-matched controls mediated in part by impaired cell proliferation, mitochondrial respiratory function, and ATP production. LC-MS/MS profiling of plasma and tissues from BAM15-treated animals revealed distribution of BAM15 in adipose, liver, and tumor tissue with low abundance in skeletal muscle.

**Conclusions:**

Collectively, these data indicate that mitochondrial uncoupling may be an effective strategy to limit proliferation of aggressive forms of breast cancer. More broadly, these findings highlight the metabolic vulnerabilities of highly proliferative breast cancers which may be leveraged in overcoming poor responsiveness to existing therapies.

**Supplementary Information:**

The online version contains supplementary material available at 10.1186/s40170-021-00274-5.

## Introduction

Breast cancer is a highly prevalent and heterogeneous malignancy with more than 275,000 new cases diagnosed annually [1]. Mortality is attributable to the aggressive, more recurrent, and highly metastatic forms, such as triple-negative (TNBC) and estrogen receptor (ER)-negative breast cancer, which respond poorly to currently available targeted drug regimens [2–7]. Notably, co-morbidities such as obesity worsen clinical prognosis and exacerbate resistance to chemotherapy [8–17]. As such, there is a critical need to develop therapies which improve survival and reduce recurrence in patients with aggressive forms of breast cancer.

We have recently demonstrated that a furazano[3,4-b]pyrazine, named BAM15 (N5,N6-bis(2-Fluorophenyl)[1,2,5]oxadiazolo[3,4-b]pyrazine-5,6-diamine), is a tolerable and bioavailable mitochondrial targeted protonophore that restores lipid metabolism and glycemic control in pre-clinical models of obesity [18, 19]. The notion that mitochondrial uncouplers have direct anti-cancer action is somewhat paradoxical in that the cellular compensation to reduced ATP synthesis is to stimulate nutrient uptake, subsequently increasing aerobic glycolysis and improving cell survival [20]. Conversely, it has been theorized that in order to meet cellular energetic demand, transient reductions in bioenergetic efficiency stimulate pyruvate flux into the mitochondria for complete oxidation, diminishing glycolytic potential and suppressing anabolic activity [21]. Uniquely, highly proliferative and metastatic breast cancer cells increasingly rely on glycolysis compared to hormone sensitive cells which impairs immune function and drives tumor growth [22]. Despite this, glycolytic inhibitors alone appear inadequate at tumor suppression, potentially selecting for more resilient cellular subpopulations [23], indicating that oxidative phosphorylation (OXPHOS) is central to cell survival [24].

Here, we present the first evidence that reducing mitochondrial coupling efficiency by BAM15 suppresses the growth of highly proliferative breast cancers. We observed that BAM15 dose-dependently reduced proliferation and migration in human TNBC MDA-MB-231 and murine Luminal B, ERα-negative EO771 cells [25]. Additionally, BAM15 increased apoptosis in MDA-MB-231 and EO771 cells and displayed comparable growth inhibition to FDA-approved chemotherapies used in the treatment of TNBC. Untargeted transcriptomic profiling of BAM15-treated MDA-MB-231 cells revealed marked downregulation of pathways required for cell division, growth, and survival, as well as energy production. BAM15 reduced tumor growth in orthotopically injected C57BL/6J mice fed a standard or high fat diet (HFD). Suppression of tumor growth in vivo was attributable to reduced proliferative capacity, increased cancer cell death, and dampened mitochondrial function. Taken together, these data support the notion that reducing bioenergetic efficiency is an effective strategy to suppress growth of aggressive breast cancers. Furthermore, these studies highlight the potential therapeutic value of leveraging bioenergetic profiles of cancer cells for targeted treatment.

## Results

### BAM15-mediated mitochondrial uncoupling reduces cell viability, proliferation, and migration in human TNBC and murine luminal B breast cancer cells

**Fig. 1:**
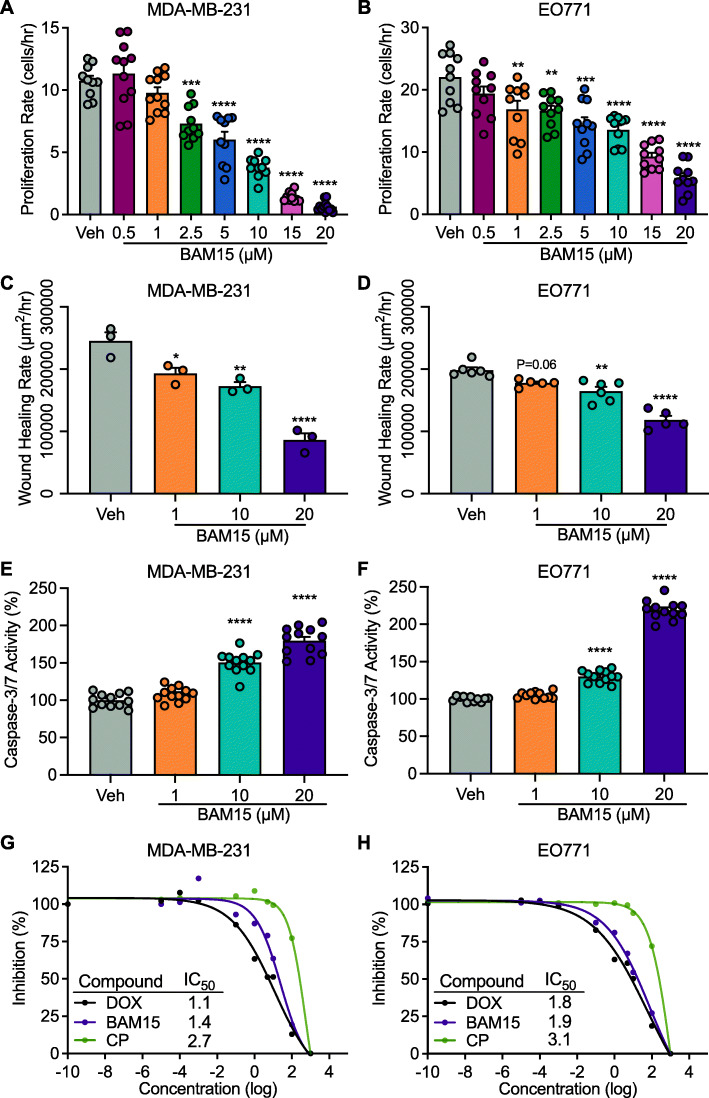
BAM15 mediated mitochondrial uncoupling reduces cell viability, proliferation, and migration in MDA-MB-231 and EO771 cells. Change in cellular proliferation over 4.5 days of continuous exposure to varying concentrations of BAM15 in **A** MDA-MB-231 (*N*=10 for Veh, 2.5, 5, and 10 1 μM BAM15, *N*=11 for 0.5 and 1 μM BAM15, *N*=12 for 15 and 20 μM BAM15) and **B** EO771 cells (*N*=10 per condition). Wound healing rate after continuous exposure to varying concentrations of BAM15 in **C** MDA-MB-231 (*N*=3 per condition) and **D** EO771 cells (*N*=6 per condition). Caspase 3/7 activity after 16-h exposure to varying concentrations of BAM15 in **E** MDA-MB-231 cells and **F** EO771 cells (*N*=12 per condition). Inhibition of cell viability (IC_50_,) after 24-h continuous exposure to varying concentrations of BAM15, doxorubicin, or cyclophosphamide in **G** MDA-MB-231 cells and **H** EO771 cells (*N*=12 per condition). Panels **A**, **B**, **C**, **D**, **E**, and **F** are shown as the mean ± SEM and were assessed by one-way ANOVA with Tukey’s multiple comparisons. **p<0.01, ***p<0.001,****p<0.0001. Abbreviations: IC_50_, half maximal inhibitory concentration

To investigate the direct anti-cancer effects of BAM15 in vitro, cell proliferation was measured over the course of 4.5 days of expansion. We found that BAM15 resulted in a dose-dependent inhibition of proliferation in MDA-MB-231 and EO771 cells compared to the vehicle (Veh) (Fig. 1A, B). MDA-MB-231 cells displayed slower proliferation and greater sensitivity to BAM15 compared to EO771 cells (Fig. 1A, B). Both cell lines displayed early inhibition of proliferation with 20 μM of BAM15 and, after about 30 h, decreased proliferation with 10 μM, and EO771 cells also show inhibition with 1 μM (Figure S1A-S1C). Given the decrease in cell proliferation by BAM15, we hypothesized that BAM15 would impair cell movement and restrict migration. We then measured two-dimensional migration for 6 h by creating a scratch wound and monitoring the number of cells wound over time (Figure S1D). We found that BAM15 resulted in a dose-dependent inhibition of cell migration of both MDA-MB-231 and EO771 cells (Fig. 1C, D). Within 2 h of BAM15 exposure, cell migration was reduced at both 10 and 20 μM, and by 3 h, the percent wound confluence was reduced with 1 μM BAM15 in MDA-MB-231 and trending towards inhibition in EO771 cells (Figure S1D-F). We then measured apoptosis in MDA-MB-231 and EO771 cells and found that after 16 h of exposure, BAM15 increased caspase 3/7 activity at doses 10 μM and greater relative to Veh (Fig. 1E, F). Finally, we evaluated the comparative efficacy of BAM15 on MDA-MB-231 and EO771 cell growth and viability to doxorubicin and cyclophosphamide, adjuvant chemotherapies used in the treatment of TNBC and other breast cancers [26]. In both lines, the IC_50_ of BAM15 was comparable to doxorubicin and more potent than cyclophosphamide (Fig. 1G, H). Taken together, these findings indicate that BAM15 suppresses the proliferation and migration of aggressive breast cancer in vitro with comparable efficacy to the standard of care pharmacotherapies.

### BAM15 reduces the expression of genes required for cellular proliferation and energy production in MDA-MB-231 cells

**Fig. 2:**
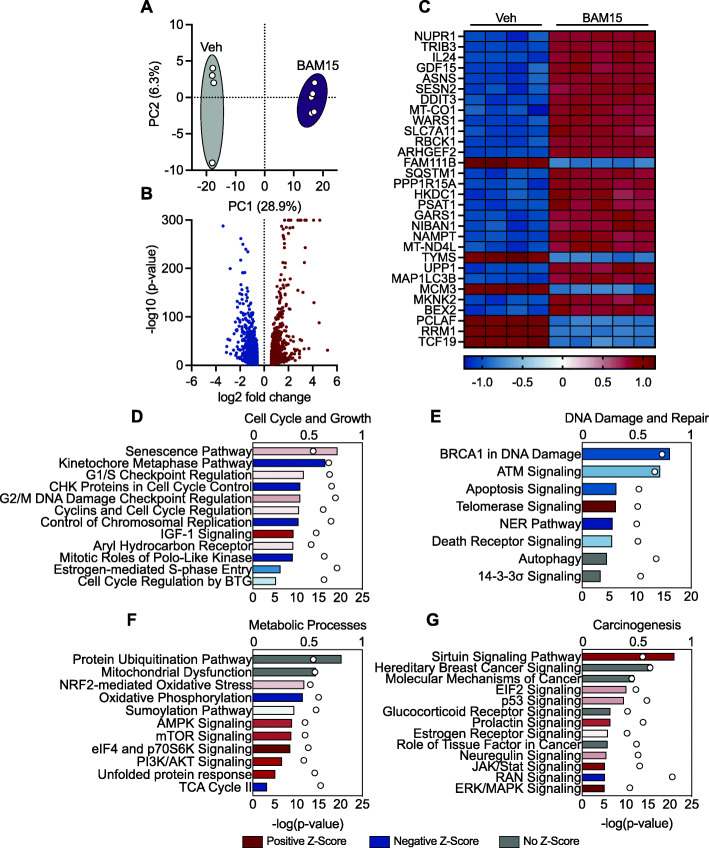
BAM15 reduces the expression of genes required for cellular proliferation and energy production in MDA-MB-231 cells. **A** Principal component analysis of cells exposed to 16 h vehicle or 20 μM BAM15. **B** Volcano plot illustration of genes differentially expressed by BAM15. **C** Heat map visualization of top 30 differentially regulated genes. Differentially regulated transcripts were filtered based on the following criteria: *q*<0.05, base mean>30, and fold change>1.5. **D**–**G** Enrichment analysis of canonical signaling pathways differentially regulated by BAM15. Circles (top *x*-axis) represent the ratio of differentially expressed genes relative to the total number of genes in a given signaling pathway. Bars (bottom x-axis) represent the probability of pathway activation or inactivation based upon differential gene expression patterns. Bar colors represent the directional *Z*-score generated from pathway enrichment analysis

To determine whether chronic exposure to BAM15 altered cell survival or metabolic fate, we performed untargeted whole transcriptome sequencing of MDA-MB-231 cells after 16 h of treatment. Principle component analysis revealed relatively similar clustering within Veh and BAM15-treated cells, which were highly dissimilar to one another (Fig. 2A). We identified 2108 transcripts that were differentially regulated by BAM15. Of the 2108 transcripts, 968 were upregulated and 1140 downregulated by BAM15 (Fig. 2B). We then visualized the top 30 differentially expressed genes in a heat map (Fig. 2C). To contextualize these findings, we performed pathway enrichment analysis on genes differentially expressed by BAM15. Canonical signaling pathways related cell survival, proliferation, energy production, and DNA damage were differentially regulated by BAM15 (Fig. 2D–G). Several transcriptional factors essential to mitochondrial membrane dynamics including *DNML1* and *OPA1* were downregulated by BAM15 treatment (Figure S2A, Table S1). Transcriptional regulators of autophagy such as *SIRT1*, *SIRT3*, *ULK1*, and *ATG2A* were upregulated in response to BAM15 treatment (Figure S2B, Table S1). Given the augmentation in transcriptional regulation of membrane functions and cellular autophagy, we then sought to evaluate expression of genes required for respiratory complex formation. Transcriptional regulation of complex I (*NDUFS1*), II (*SDHB*), III (*UQCRC2*), and V (*ATP5F1A*) was decreased by BAM15 treatment, whereas complex IV (*MT-CO1*) was increased (Figure S2C, Table S1). Genes essential to the regulation of cellular energy metabolism were largely unchanged with the exception of three functional domains on AMPK (*PRKAB1*, *PRKAB2*, *PRKAG1*), which were increased and *AKT1* which was decreased following BAM15 treatment (Figure S2D, Table S1). Transcriptional regulation of glycolysis, the TCA cycle, and lipid metabolism were largely downregulated with BAM15 (Figure S2E-I, Table S1). Exceptions included mitochondrial *PEPCK (PCK2)* and *GOT1*, both of which are commonly increased during low-glucose conditions as a survival response to stress (Figure S2E-F, Table S1). Furthermore, all TCA cycle gene expression was decreased (Figure S2G-H, Table S1). Notably, genes required for fatty acid transport (*CPTII*) into the mitochondria were increased while other transporters (*SLC25A20*) were decreased (Figure S2I, Table S1). Genes essential to insulin receptor signaling such as *IRS2*, *FOXO3*, and *PIK3* were elevated but without an increase in *AKT* expression (Figures S2J, Table S1). Genes involved in early steps of the pentose phosphate pathway such as *G6PD*, *H6PD, PGLS*, and *RBKS* were increased while others were decreased or remained unchanged (Figure S2K, Table S1). Taken together, these findings indicate the mitochondrial uncoupling by BAM15 reduces transcriptomic signatures associated with cell survival and energy production.

### BAM15 reduces OXPHOS and glycolytic capacity via ΔΨm destabilization in MDA-MB-231 and EO771 cells

**Fig. 3:**
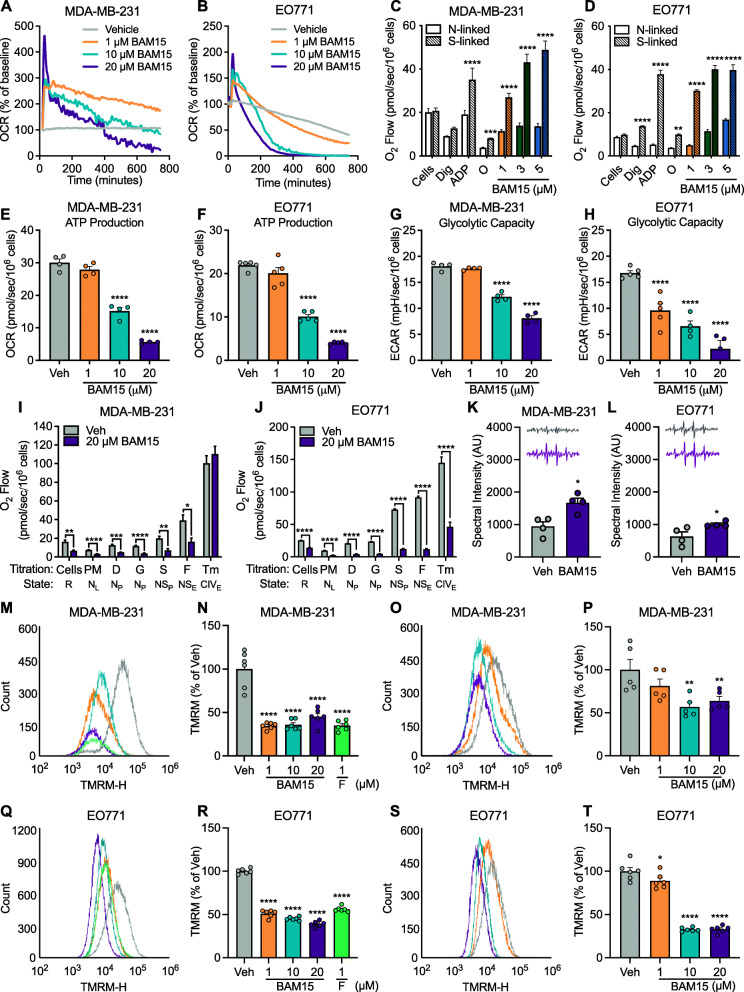
BAM15 reduces OXPHOS and glycolytic capacity via ΔΨm destabilization in MDA-MB-231 and EO771 cells. Oxygen consumption rates following acute injection of varying concentrations of BAM15 in **A** MDA-MB-231 (Veh *N*=5, 1 μM BAM15 *N*=5, 10 μM BAM15 *N*=3, 20 μM BAM15 *N*=4) and **B** EO771 cells (Veh *N*=6 per condition). Respiration supported by malate, pyruvate, and glutamate (N-linked), or succinate (S-linked) in the presence of ADP, oligomycin, and varying concentrations of BAM15 in digitonin-permeabilized cells in **C** MDA-MB-231 (*N*=6 per condition) and **D** EO771 cells (*N*=4 per condition). ATP-linked respiration in **E** MDA-MB-231 and **F** EO771 cells (*N*=4 per condition). Maximal glycolytic rate in **G** MDA-MB-231 and **H** EO771 cells the presence of glucose and oligomycin (*N*=4 per condition for MDA-MB-231; *N*=5 per condition for EO771 cells) following 16-h exposure to vehicle or varying concentrations of BAM15. Respiration supported by malate, pyruvate, glutamate, and succinate in the presence of ADP, FCCP, and ascorbate/TMPD after 16-h exposure to vehicle or 20 μM BAM15 in digitonin-permeabilized **I** MDA-MB-231 (*N*=8 per condition) and **J** EO771 cells (*N*=4 per condition). Superoxide production following 16-h exposure to vehicle or 20 μM BAM15 in **K** MDA-MB-231 (*N*=4 per condition) and **L** EO771 cells (*N* =4 per condition). **M** Representative flow cytometry plot, and **N** quantification of TMRM florescence after acute exposure to varying concentrations of BAM15 or FCCP (*N*=6 per condition) and **O**, **P** chronic exposure to varying concentrations of BAM15 (*N*=5 per condition) in MDA-MB-231 cells. **Q** Representative flow cytometry plot, and **R** quantification of TMRM florescence after acute exposure to varying concentrations of BAM15 or FCCP (*N*=6 per condition) and **S**, **T** chronic exposure to varying concentrations of BAM15 (*N*=6 per condition) in EO771 cells. Data are shown as the mean ± SEM. *p<0.05, **p<0.01, ***p<0.001, ****p<0.0001. Panels **C**, **D**, **E**, **F**, **G**, **H**, **K**, **L**, **N**, **P**, **R**, and **T** were assessed by one-way ANOVA with Tukey’s multiple comparisons. Panels **A**, **B**, **I**, and **J** were assessed by two-way repeated measures ANOVA with Sidak’s multiple comparisons. Abbreviations: OCR, oxygen consumption rate; ECAR, extra-cellular acidification rate; Dig, digitonin; ADP, adenosine 5′-diphosphate; O, oligomycin; PM, pyruvate and malate; G, glutamate; S, succinate; FCCP, Carbonyl cyanide-4-(trifluoromethoxy)phenylhydrazone; TMPD, tetramethyl-p-phenylene diamine; TMRM, tetramethylrhodamine methyl ester

BAM15 is a protonophore mitochondrial uncoupler [27]; therefore, we evaluated the extent to which alterations in mitochondrial function may contribute to reduced proliferation and survival of aggressive breast cancer. In MDA-MB-231 and EO771 cells, BAM15 rapidly increased oxygen consumption in a dose-dependent manner (Fig. 3A, B and Figure S3A-B). The oxygen consumption rate declined more rapidly after exposure to BAM15 at 10 and 20 μM doses and after 12 h (MDA-MB-231 cells) or 3 h (EO771 cells) was decreased relative to Veh (Fig. 3A, B). Despite markedly increased oxygen consumption, exposure to BAM15 decreased time to 50% reduction in uncoupling activity (Fig. 3A, B). We then investigated if the acute increase in oxygen consumption occurred in a substrate dependent manner. In both MDA-MB-231 and EO771 cells, BAM15 increased leak respiration supported by pyruvate + malate (N-linked; Complex I) and to a greater extent succinate (S-linked; Complex II) (Fig. 3C, D). In MDA-MB-231 cells, OXPHOS supported by succinate was limited by phosphorylation, whereas N-linked substrates were limited by oxidation (Fig. 3C, Figure S3C). In EO771 cells, both N- and S-linked OXPHOS were limited by oxidation (Fig. 3D, Figure S3D). In both MDA-MB-231 and EO771 cells, the oxidation of succinate was greater than pyruvate + malate (Figure S3C-D). We then evaluated whether long-term exposure to BAM15 altered respiratory capacity and ATP production. In intact MDA-MB-231 and EO771 cells, 16-h exposure to BAM15 diminished ATP production (Fig. 3E, F) and glycolytic capacity (Fig. 3G, H) in a dose-dependent manner. Additionally, the rate of intact cellular respiration and electron transfer decreased dose-dependently compared to Veh (Figure S3E-H). We then further evaluated mitochondrial coupling control in digitonin-permeabilized MDA-MB-231 and EO771 cells after chronic exposure to BAM15 (Fig. 3I, J) and found that routine respiration as well as N and NS-linked OXPHOS were decreased relative to Veh (Fig. 3I, J). MDA-MB-231 and EO771 cells were minimally responsive to stimulation with FCCP, indicating defective ET capacity (Fig. 3I, J). Additionally, complex IV (CIV) activity was decreased in EO771 but intact in MDA-MB-231 cells (Fig. 3I, J).

We then postulated that mitochondrial dysfunction facilitates generation of reactive oxygen species (ROS), which in turn may exacerbate cellular decline [28]. We observed that prolonged exposure to BAM15 increased superoxide production in MDA-MB-231 and EO771 cells (Fig. 3K, L), consistent with decreased respiratory function. Based upon our observations that prolonged exposure to BAM15 impairs mitochondrial function, increases ROS production, and that the vast majority of the proton motive force is mediated by mitochondrial membrane potential (ΔΨm) [29], we determined the acute effects of BAM15 on ΔΨm and whether or not chronic exposure limits ΔΨm recovery (Fig. 3M–T). We found that acute exposure to BAM15 dampened ΔΨm by more than 50–60% in both MDA-MB-231 and EO771 cells (Fig. 3M, N and Q, R) which saturated at 1 μM in MDA-MB-231 (Fig. 3M, N) but was dose dependent in EO771 cells (Fig. 3Q, R). After 16-h exposure to BAM15, ΔΨm nearly recovered at 1 μM (Fig. 3O, P and S, T) while remaining significantly damped by upwards of 40% at the 10 and 20 μM doses in the MDA-MB-231 cells (Fig. 3O, P) and upwards of 70% in the EO771 cells (Fig. 3S, T). Taken together, BAM15-mediated uncoupling and subsequent mitochondrial membrane depolarization in vitro results in respiratory failure and superoxide production by limiting ATP production from S-linked OXPHOS and glycolysis.

### BAM15 suppresses tumor growth in C57BL/6J mice

**Fig. 4:**
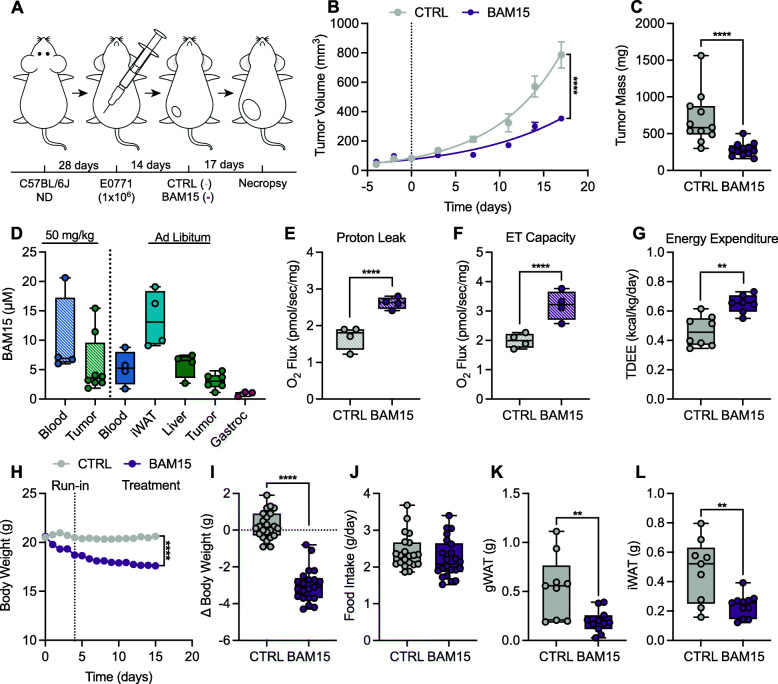
BAM15 suppresses tumor growth in lean C57BL/6J mice. **A** Schematic illustration of the experimental design. **B** Tumor volumes over the treatment period and **C** terminal tumor mass (CTRL *N*=11, BAM15 *N*=12). **D** BAM15 concentrations in plasma (*N*=4) and tumor (*N*=8) 1 h after an acute gavage of 50 mg/kg of BAM15 at time of necropsy and plasma and tissue after ad libitum access to BAM15 diet (plasma, iWAT, and liver *N*=4, tumor *N*=6, and gastrocnemius *N*=3). **E** Proton leak and **F** ET capacity in tumors 1 h after an acute gavage of 50 mg/kg of BAM15 (N=4 per condition). **G** Total daily energy expenditure in mice over the last 7 days of treatment (CTRL *N*=8, BAM15 *N*=7). **H** Body weight over the treatment period and **I** change in body weight from treatment start (CTRL *N*=23 and BAM15 *N*=21). **J** Food intake averaged over the treatment period (CTRL *N*=21 and BAM15 *N*=22). **K** Terminal gWAT weight and **L** iWAT weight (CTRL *N*=9, BAM15 *N*=11). Data are shown as a box (mean ± 5–95% CI) and whiskers (minimum to maximum) except for **B** and **H** which are displayed as the mean ± SEM. *p<0.05, **p<0.01, ***p<0.001, ****p<0.0001. Panels B and H were assessed by two-way repeated measures ANOVA with Tukey’s multiple comparisons. Panels **C**, **D**, **E**, **F**, **G**, **I**, **J**, **K**, and **L** were assessed by one-way ANOVA with Tukey’s multiple comparisons. Panel **H** was assessed by extra sum-of-squares *F* test. Abbreviations: CTRL, control; TDEE, total daily energy expenditure; gWAT, gonadal white adipose tissue; iWAT, inguinal white adipose tissue; gastroc, gastrocnemius

Based upon our observations that BAM15 limits cell survival in vitro, we sought to perform pre-clinical validation by orthotopically injecting EO771 cells into immunocompetent female C57BL/6J mice (Fig. 4A). Two weeks after injection, mice were randomized to 2.5 weeks of control (CTRL; standard diet) or BAM15 (BAM15; 0.1% w/w in a standard diet). BAM15 reduced tumor volume by day 6 relative to CTRL which persisted throughout the duration of treatment (Fig. 4B). Consistently, BAM15 reduced tumor mass relative to CTRL (Fig. 4C). To confirm that tumor mitochondria were uncoupled in vivo, mice received a 50 mg/kg oral gavage of BAM15 and were analyzed for respiratory function 1 h post gavage. We determined distribution of BAM15 by LC-MS profiling of plasma and tumor and demonstrated the presence of BAM15 in both plasma and tumor 1 h after BAM15 gavage (Fig. 4D). We observed that leak respiration and maximal electron transfer supported by N- and S-linked substrates in the presence of oligomycin were both increased after BAM15 gavage (Fig. 4E, F). Plasma and organ samples from ad libitum BAM15-treated mice revealed distribution in iWAT, liver, tumor, and to a lesser extent skeletal muscle (Fig. 4D). Body temperature was modestly decreased with BAM15 (Figure S4A). Similar to previous reports of tolerability and histopathology of BAM15 in male mice [18, 19], we did not detect signs of toxicity or organ damage with BAM15 treatment (Figure S4B-D). Given BAM15’s primary effects as a mitochondrial uncoupler, we then determined whether metabolic rate was altered by treatment. We observed that BAM15 increased energy expenditure relative to control animals with a slight decrease in the dark phase respiratory quotient (RQ) and no change in light phase RQ or locomotion (Fig. 4G and Figure S4E-G). Similar to previous reports in mice with diet-induced obesity [18, 19], BAM15-treated animals displayed reduced body weight, but similar food and water intake compared to CTRL (Fig. 4H–J and Figure S4H). Reductions in body weight were attributable to reduced fat mass compared to CTRL (Fig. 4K, L).

**Fig. 5:**
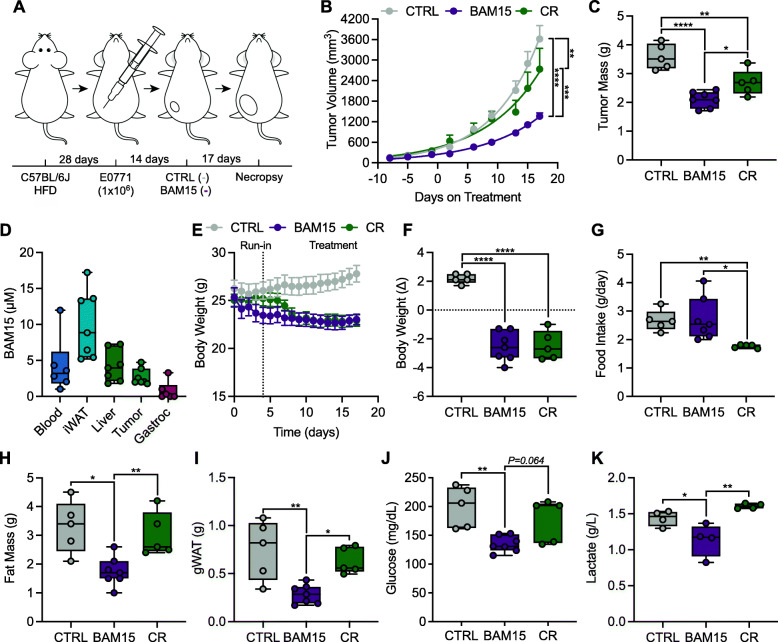
BAM15 suppresses tumor growth in C57BL/6J mice fed a high fat diet. **A** Schematic illustration of the experimental design. **B** Tumor volumes over the treatment period and **C** terminal tumor mass (CTRL *N*=5, BAM15 *N*=7, and CR *N*=5). **D** BAM15 distribution in tissue and plasma at time of necropsy (*N*=6 for plasma, *N*=7 for tissues). **E** Body weight over the treatment period and **F** change in body weight from treatment start (CTRL *N*=5, BAM15 *N*=7, and CR *N*=5). **G** Food intake averaged over the treatment period (CTRL *N*=5, BAM15 *N*=7, and CR *N*=5). **H** Total fat mass (CTRL *N*=5, BAM15 *N*=7, and CR *N*=5). **I** Terminal gWAT weight (CTRL *N*=5, BAM15 *N*=7, and CR *N*=5). **J** Plasma glucose and **K** lactate concentrations at time of necropsy (*N*=4 per group). Data are shown as box (mean ± 5-95% CI) and whiskers (minimum to maximum) with exception to panels **B** and **E** which are displayed as mean ± SEM. *p<0.05, **p<0.01, ***p<0.001, ****p<0.0001. Panels **B** and **E** were assessed by two-way repeated measures ANOVA with Tukey’s multiple comparisons. Panels **C**, **D**, **F**, **G**, **H**, **I**, **J**, and **K** were assessed by one-way ANOVA with Tukey’s multiple comparisons. Abbreviations: CTRL, control; CR, calorie restriction; gWAT, gonadal white adipose tissue; gastroc, gastrocnemius

Given the protective effects of BAM15 on weight and fat gain and the known exacerbation of breast cancer progression by obesity, we performed an additional study whereby mice with diet-induced obesity were randomized to 2.5 weeks of high-fat diet (HFD) control (CTRL; 60% kcal from HFD), BAM15 (BAM15; 0.1% w/w in HFD), or calorie restriction (CR; restriction of HFD) (Fig. 5A). Overall, high fat feeding markedly accelerated and exacerbated tumor growth and mass (Fig. 5B, C). However, BAM15 reduced tumor volume by day 6 relative to CTRL and day 13 relative to CR which persisted throughout the duration of treatment (Fig. 5B). Consistently, BAM15 reduced tumor weights relative to both CTRL and CR (Fig. 5C). LC-MS profiling of plasma and organ samples from BAM15-treated mice revealed distribution in iWAT, liver, tumor, and to a lesser extent skeletal muscle (Fig. 5D). BAM15 and CR animals displayed equally reduced body weight compared to CTRL (Fig. 5E, F). Food intake was similar between CTRL and BAM15 mice, and weight matching required 40% CR (Fig. 5G). Additionally, BAM15 reduced fat mass compared to CTRL and CR (Fig. 5H). Additionally, gWAT (Fig. 4I) mass was decreased by BAM15 relative to both CTRL and CR. We observed metabolic improvements in the BAM15 group that were not achieved in the CR group. Glucose concentrations in the BAM15-treated animals were significantly lower than CTRL mice (Fig. 5J), and BAM15-treated animals had lower lactate levels compared to both CTRL and CR (Fig. 5K). Taken together, these data indicate that BAM15 slows tumor growth in mice fed a standard or high fat diet.

### BAM15 reduces tumor growth in vivo by inhibiting proliferation and reducing mitochondrial function

**Fig. 6:**
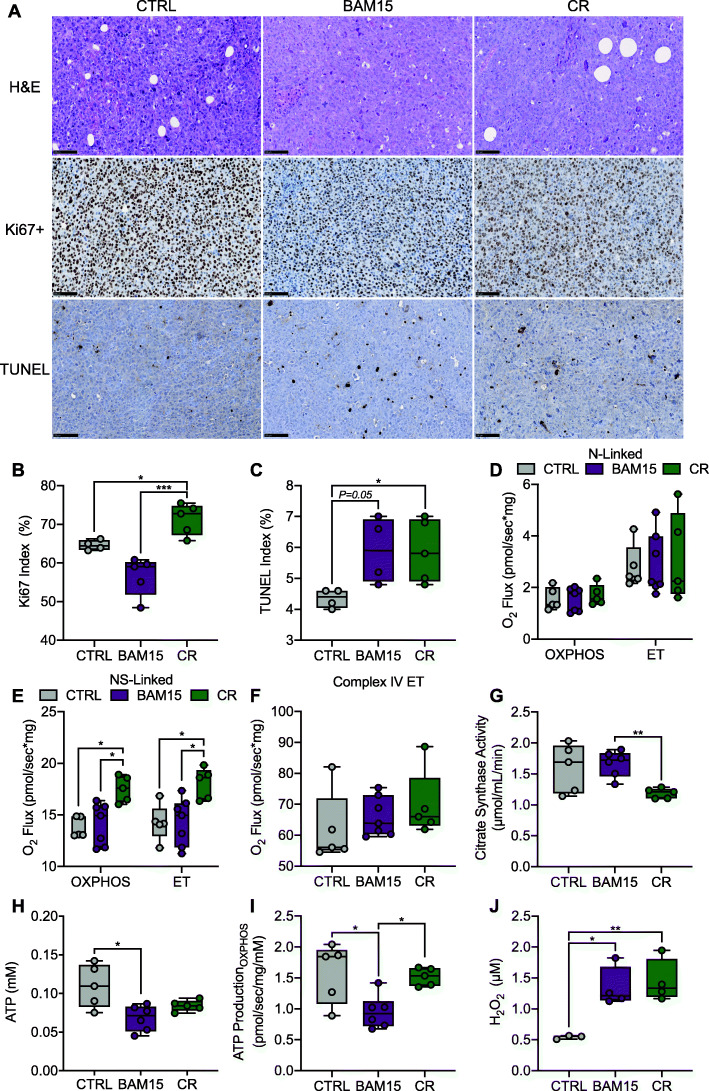
BAM15 reduces tumor growth in vivo by limiting proliferation and mitochondrial function. **A** Representative H&E (scale = 100 μm), Ki67+ (scale = 100 μm), and TUNEL (scale = 100 μm) staining and quantification of **B** Ki67+ and **C** TUNEL staining in tumor sections (CTRL *N*=4, BAM15 *N*=5, CR *N*=5). **D** ADP stimulated respiration (OXPHOS) and FCCP stimulated respiration (ET) supported by pyruvate, malate, glutamate (N-linked), and **E** succinate (NS-linked), (CTRL *N*=5, BAM15 *N*=7, CR *N*=5). **F** FCCP stimulated respiration in the presence of ascorbate/TMPD (Complex IV ET) (CTRL *N*=5, BAM15 *N*=7, CR *N*=5). **G** Enzymatic activity of citrate synthase (CTRL *N*=5, BAM15 *N*=6, CR *N*=5). **H** ATP content (CTRL *N*=5, BAM15 *N*=6, CR *N*=5) and **I** ATP production derived from NS-linked OXPHOS (CTRL *N*=5, BAM15 *N*=6, CR *N*=5). **J** Hydrogen peroxide (H_2_O_2_) activity in tumor homogenates (CTRL *N*=3, BAM15 *N*=4, CR *N*=4). Data are shown as the mean ± SEM. *p<0.05, **p<0.01, ***p<0.001. Panels **B**, **C**, **D**, **E**, **F**, **G**, **H**, **I**, and **J** were assessed by one-way ANOVA with Tukey’s multiple comparisons. Abbreviations: CTRL, control; CR, calorie restriction; H&E, Hematoxylin and Eosin; Hm, homogenate; PM, pyruvate and malate; D, ADP; G, glutamate; c, cytochrome c; S, succinate; U, uncoupler (FCCP); Rot, rotenone; AmA, antimycin A; As, ascorbate; TMPD, tetramethyl-p-phenylenediamine dihydrochloride; Azd, sodium azide; H_2_O_2_, hydrogen peroxide

Based on our observation that BAM15 reduces cell proliferation in vitro, we then conducted a histological evaluation in CTRL, BAM15, and CR tumors. We found that most neoplasms were densely cellular and consisted of poorly differentiated infiltrative carcinomas with delicate stroma and minimal inflammatory reaction (Fig. 6A). All tumors displayed high expression of the Ki67 proliferation marker, and notably, there was variability in staining localization with possible differences in phases of the cell cycle (Fig. 6A). We found that tumors in BAM15-treated animals exhibited reduced Ki67+ staining compared to both CTRL and CR (Fig. 6A, B). Additionally, we measured cell death using TUNEL staining that binds to DNA fragments. Most neoplasms contained large areas of necrosis with saturated TUNEL positivity, and thus, we quantified TUNEL staining from non-necrotic regions. We found that CR-treated animals had increased cell-death and BAM15-treated animals had a trend towards increased cell-death in these areas compared to CTRL (Fig. 6A, C). Since reduced cell proliferation was linked to impaired mitochondrial function in vitro, we then measured OXPHOS and ET capacity in tumor homogenates ex vivo. Similar to our in vitro studies, tumors displayed reliance on S-linked flux for oxidative phosphorylation. N-linked (PM or PMG) oxidation was minimal, limited by OXPHOS, and did not differ between groups (Fig. 6D). NS-linked OXPHOS and ET (Fig. 6E) capacity were unchanged between CTRL and BAM15 but decreased by BAM15 relative to CR. Complex IV ET did not differ between groups (Fig. 6F). Interestingly, citrate synthase activity was reduced in CR relative to BAM15 (Fig. 6G), further supporting a reduction in NS-linked OXPHOS and ET by BAM15. BAM15 reduced tumor ATP content relative to CTRL (Fig. 6H). Furthermore, the contribution of OXPHOS to the pool of ATP was reduced by BAM15 relative to both CTRL and CR, indicating overall worsening of mitochondrial function (Fig. 6I). Since BAM15 increased ROS production in vitro, we then evaluated H_2_O_2_ from tumor homogenates. Tumor H_2_O_2_ production was increased by both BAM15 and CR relative to CTRL (Fig. 6J). Taken together, these data indicate that BAM15-mediated reduction in bioenergetic efficiency limits tumor growth in vivo by suppressing cell growth and impairing mitochondrial function in a weight-independent manner.

## Discussion

Tumor metabolism and energy reprogramming has recently regained traction as a critical area of focus in cancer research [30]. Cancer cells often exhibit altered mitochondrial function, including mitochondrial DNA mutations, augmented energy metabolism, elevated reactive oxygen species (ROS) generation, and increased mitochondrial membrane potential [31, 32]. The term “Mitocans” encompasses the broad range of anti-cancer agents that act via cancer cell mitochondrial destabilization, and uncouplers of oxidative phosphorylation have recently been included as promising new agents [33]. Here, we provide evidence that suppression of bioenergetic efficiency by BAM15, a mitochondrial targeted small molecule protonophore, inhibits cell growth and reduces survival of multiple models of aggressive breast cancer. BAM15 treatment was safe and did not alter function or structure in non-cancerous organ systems, indicating that treatment selectively restricted growth of cancer cells.

Numerous chemical uncoupling agents, including carbonylcyanide m-chlorophenylhydrazone (CCCP), carbonyl cyanide p-trifluoromethoxyphenylhydrazone (FCCP), and 2,4-dinitrophenol (DNP), have shown some promise in arresting cancer progression, but with varying efficacies [34]. However, these compounds can result in off-target effects such as cellular membrane depolarization and systemic hyperthermia, and some have not been well-tolerated in mice or humans, ultimately limiting clinical application [35]. More recently, Wang et al. demonstrated efficacy for a highly tolerable liver-specific controlled-release mitochondrial protonophore (CRMP) to reduce tumor growth in multiple pre-clinical models of colon cancer [36]. CRMP was found to reduce tumor growth primarily by enhancing suppression of hepatic glucose production and reducing hyperinsulinemia [36], essential mediators of obesity-associated cancer progression [37]. Additionally, the FDA-approved anthelmintic drug niclosamide displays uncoupling activity and limits tumor growth in numerous preclinical cancer models [38]. However, phase 1 trials of niclosamide were futile as plasma concentrations at the maximum tolerated dose did not reach the anti-cancer therapeutic threshold [39]. In our studies, BAM15 displayed direct anti-cancer activity in vitro and in vivo while also improving markers of systemic metabolic health, indicating that bioenergetic efficiency is intimately linked to the survival of aggressive breast cancers. This is supported in part by a previous report demonstrating that BAM15 promotes apoptosis in combination with a mitogen-activated protein kinase (MAPK) pathway inhibitor in melanoma cells [40, 41].

Despite limited research on mitochondrially targeted chemical protonophores, uncoupling has been thoroughly investigated in the context of the endogenous uncoupling proteins (UCPs) [42]. UCP’s dynamically regulate bioenergetic efficiency by regulating proton transport in response to free fatty acids resulting in ΔΨm depolarization and impact cancer growth and survival in a model-dependent and tissue-specific manner [43]. For example, UCP2 expression is upregulated in several tumor types as well as some chemo-resistant cell lineages [44, 45]. Overexpression of UCP1 in brown adipose tissue and UCP3 in skeletal muscle exacerbates weight loss in models of cancer cachexia [46, 47]. Conversely, overexpression of UCPs (1, 2, and 3) in models of aggressive breast cancer impairs tumor growth in mice [48]. Importantly, the degree of uncoupling and sustained ΔΨm depolarization appears to have a distinct role in the treatment of cancer. Notably, a study by Chonghaile et al. demonstrated that clinical response to chemotherapy in patients was correlated with the degree to which the tumor mitochondria were depolarized [49]. These data demonstrate the anti-tumor potential of mitochondrial-targeted compounds that adequately stress mitochondria through substantial and sustained depolarization.

Our studies show that BAM15-mediated uncoupling results in sustained ΔΨm depolarization, limits cancer cell and tumor ATP-related OXPHOS, increases superoxide production, increases caspase-3/7 activity, and ultimately suppresses tumor progression. Dramatic and sustained reductions in ATP production and availability are well-characterized determinants of cell death [50]. Furthermore, mitochondria can initiate cell death through release of cytochrome c into the cytosol which activates a number of caspase proteases to execute programmed cell death and are inducible by ΔΨm depolarization and increased oxidative stress [51–54]. Several aggressive sub-types of breast cancer, including triple negative, are rich in oxidants which drive DNA mutations to ensure cell survival [55]. Interestingly, exposure to either antioxidants, such as flavonoids and α-tocopherol, or oxidative stressors, such as chemotherapy and radiation, have been shown to limit cancer cell progression [56, 57]. By limiting redox potential, antioxidants also prevent signal transduction via growth-initiating pathways; however, in the presence of a mitochondrial uncoupler, a considerable rise in oxidative stress may limit survival by activation of autophagy and apoptosis [54].

We determined that succinate is a primary substrate for OXPHOS in aggressive breast cancers and that N-linked substrates such as pyruvate and malate marginally contribute to oxidation. This is further supported by a number of immortalized breast cancer cell lines containing genetic mutations and functional defects in Complex I [58, 59]. The importance of OXPHOS as a source for ATP production and cell survival is demonstrated by the efficacy of inhibitors of complex I such as metformin [60], complex II by the antineoplastic compound lonidamine [61], or complex III as demonstrated by the FDA-approved Atovaquone [62]. Importantly, NS-linked OXPHOS and ET capacity was decreased after prolonged exposure to BAM15. Tumors exposed to BAM15 were less bioenergetically efficient which was expected as mitochondrial uncoupling decreases caloric efficiency as an on-target mechanism of action. More notably, BAM15-treated cells and tumors were unable to adequately supply ATP through either OXPHOS or glycolysis, ultimately diminishing ATP producing potential and subsequently content. This observation is somewhat paradoxical in that inhibitors of oxidation, such as rotenone or metformin, can stimulate compensatory glycolysis to enhance cell survival [63, 64]. Since the endothermic step of glycolysis requires ATP investment, glycolytic compensation in response to such respiratory inhibitors indicates that ATP production is not sufficiently restricted. This observation is unsurprising bioenergetically as ATP generation is a highly conserved process supported by multiple dehydrogenases. Conversely, mitochondrial uncouplers, such as BAM15, directly limit ATP production rather than inhibiting specific dehydrogenase activity. As such, uncoupling agents may offer broader therapeutic value by preventing compensatory increase in either glycolytic or OXPHOS activity and ultimately limiting maladaptation.

## Conclusions

Taken together, we show that BAM15 limits cancer cell proliferation and migration while increasing apoptosis. On a broader scale, these studies provide the conceptual basis for determining the therapeutic potential of mitochondrial uncouplers in the treatment of aggressive breast cancers. Furthermore, they demonstrate a proof-of-concept for leveraging the unique bioenergetic profiles of cancer cells in the screening and identification of novel antineoplastic agents. Future studies are required to determine synergy of mitochondrial uncouplers, such as BAM15, with conventional chemotherapeutic regimens for treatment of aggressive breast and other forms of cancer.

## Materials and methods

### Tissue culture

Human MDA-MB-231 (ATCC, Manassas, VA, USA, passages 4–8) and murine E0771 cells (CH3 BioSystems, Amherst, NY, USA, passages 4–8) were cultured in growth media containing high glucose DMEM (4.5 g/L D-glucose) supplemented with 10% fetal bovine serum (FBS), 1% penicillin-streptomycin (100 U/mL), and 0.2% amphotericin B. E0771 cells were used for the in vivo allograft study and were grown in RPMI 1640 supplemented with 10 mM HEPES, 10% fetal bovine serum (FBS), 1% penicillin-streptomycin (100 U/mL), and 0.2% amphotericin B. All cells were maintained in a 37°C humidified incubator with 5% CO_2_.

### Animal care

Four-week-old female C57BL/6J mice were purchased at weaning from the Jackson Laboratory (Stock #000664, Bar Harbor, ME, USA). All mice were single-housed and maintained in a conventional animal facility at 21–22°C at a relative humidity of 50±10% on a 12-h light:dark cycle from time of weaning. Mice were fed a diet consisting of 60% kcal from fat, 20% kcal from protein, and 20% kcal from carbohydrate (#D12492, Research Diets, New Brunswick, NJ, USA) or 16% kcal from fat, 20% kcal from protein, and 64% kcal from carbohydrate (#D11112201, Research Diets, New Brunswick, NJ, USA) according to the study design. Animals were given ad libitum access to food (maintaining approximately 50 g of food in the hopper) and water unless indicated. Prior to treatment allocation or experimentation, each animal was weighed weekly and body temperature was recorded via infrared thermometry during weighing (Fluke 572-2 Infrared Thermometer; Fluke, Everett, WA, USA).

### Method details

#### In vitro cell studies

##### Proliferation

Kinetic proliferation was measured using label-free cell counting. MDA-MB-231 and EO771 cells were seeded into 96-well plates at 2.0 × 10^3^ cells/well and treated with varying concentrations of BAM15 or vehicle. Cell culture plates were transferred to a Cytation™ 5 Cell Imaging Multi-Mode Reader (BioTek Instruments, Winooski, VT) and imaged twice a day over a 5-day period. Two high-contrast brightfield images were captured at each time point: an in-focus image was used for qualitative evaluation and a defocused image for cell counting. Image preprocessing, object masking, and analysis parameters for cell identification and counting were set according to the manufacturer’s guide and cell counts were generated using Gen 5 Analysis software. Proliferation rates were determined by regression analysis of linear regions of the proliferation curve (0–85 h).

##### Migration

Kinetic migration was determined using a label-free image-based 2D scratch wound healing experiment with live-cell microscopy [65]. MDA-MB-231 and EO771 cells were seeded into 24-well plates at 1.0 × 10^4^ cells/well and grown to confluency. Once confluent, a thin “wound” was introduced by scratching with a pipette tip. Cells were then gently washed in PBS and treated with varying concentrations of BAM15 or vehicle (DMSO) in serum-free media to avoid proliferation. Cell culture plates were transferred to a Cytation™ 5 Cell Imaging Multi-Mode Reader (BioTek Instruments, Winooski, VT) and imaged over a 24-h period. Cellular analysis was performed on 4x phase contrast images captured over the incubation period. We quantified progression of cell migration as a function of time in the kinetic experiment using a series of data reduction steps outlined below. Cellular analysis parameters were used to develop masks to identify cells and used to calculate the total cell area (Sum Area)t which includes confluent cell space on both sides of the wound as well as individual cells that move into the wound over time. To account for intra- and inter-experiment variability in wound width, we used the following equation: *W*_t_ = *P*_A_ − (Sum Area)_t_*P*_L_, where *W*_t_ is the wound width (μm) as a function of time, *P*_A_ is the image plug area (μm2), *P*_L_ is the image plug length (μm), and the (Sum Area)_t_ is the total area (μm^2^). The average wound width at the first kinetic time point was used to calculate the percent wound confluence: % confluency = (Sum Area)_t_ −*P*_t_ (*P*_W_−*P*_t=0_)/ *P*_L_ *W*_t=0_, where *W*_t=0_ is the average wound width (μm) at the first time point, *P*_W_ is the image plug width (μm), *P*_t=0_ is the image plug width at time 0, and P_L_ and the (Sum Area)_t_ are defined above. We calculated the highest slope of the actual wound area curve to provide the maximum wound healing rate in μm^2^ per minute.

#### Cell viability and IC_50_

MDA-MB-231 and EO771 cells were seeded into black-walled 96-well plates at 2.0 × 10^4^ cells/well and incubated until 90% confluent. After treatment with doxorubicin, cyclophosphamide, or BAM15 at various concentrations (0–100 μM) for 24 h the cellular viability was measured by the 3-(4,5-dimethylthiazol-2-yl)-5-(3-carboxymethoxyphenyl)-2-(4-sulfophenyl)-2H-tetrazolium (MTS) assay using a CellTiter 96®AQueous Assay kit (Promega. Madison, WI). Saline and 0.1% Triton X-100 were used as a positive (100% viability) negative control (0% viability), respectively. In brief, a 1:5 dilution of the MTS reagent in complete medium (100 uL/well) was added directly to the adherent cells and then incubated at 37°C for 4 h. Absorbance was recorded at 490/630nm every hour using a Cytation 5 Cell Imaging Multi-Mode Reader (Biotek, Winooski, VT). MTS assay data were analyzed using a non-linear fit with variable slope evaluation in Graphpad Prizm® Software, Version 8 for Windows. IC_50_ concentrations were calculated using the 2-parameter Hill equation varying between 0 and 100 [66].

#### Caspase 3/7 activity

Caspase 3/7 activation was determined by commercially available luminescent luciferase assay according to manufacturer’s protocol (Promega). Briefly, MDA-MB-231 and EO771 cells were seeded at 2.0 × 10^4^ cells/well into black walled 96-well plate. Once cells reached 90% confluency, media was removed, and cells were treated with varying concentrations of BAM15 or vehicle for 24 h in growth media. Following treatment, caspase reagent was added directly to the treatment media and the luminescent intensity was detected on a microplate luminometer (BioTek Instruments). Data are expressed as the fold induction of caspase 3/7 activity relative to vehicle (0.01% DMSO).

#### Total RNA extraction

MDA-MB-231 cells were grown to approximately 80% confluence and treated with a vehicle (0.01% DMSO) or 20 μM BAM15 for 16 h. After treatment, cells were lysed and total RNA was extracted using TRIzol Reagent (Thermo Fisher, Waltham, MA) per manufacturer protocol. RNA was solubilized in RNase-free water. RNA yield and purity were quantified by measuring absorbance at 230, 260, and 280 nm using a microvolume spectrophotometer (NanoDrop 8000; ThermoFisher). Solubilized RNA was stored at −80°C for downstream applications.

#### RNA sequencing

RNA was normalized to 150 ng/μL in nuclease-free water. RNA integrity was assessed using an Agilent Bioanalyzer 2100. Libraries were constructed and sequenced using Lexogen QuantSeq. Briefly, library generation was performed using an oligodT primer, and double-stranded cDNA was purified with magnetic beads. Libraries were amplified using PCR, and transcripts were forward-sequenced at 75 bp using NextSeq 500 (Illumina). BlueBee software was used to analyze alignment and the DESeq2 package in R was used for differential expression analysis. Pathway enrichment was analyzed by Ingenuity Pathway Analysis software. Differentially regulated transcripts were filtered based upon the following criteria: *q*<0.05, base mean>30, and fold change>1.5. RNA sequencing was deposited in the Gene Expression Omnibus (GEO) repository under the accession number GSE161502.

#### Mitochondrial respiratory kinetics

Oxygen consumption dynamics were assessed by real-time, intact cell respirometry (Seahorse XFe24; Agilent). MDA-MB-231 and EO771 cells were seeded at 1.0 × 10^4^ cells/well in an XFe 24-well plate (Agilent). Following expansion, media was removed, and cells were incubated at 37°C for 1 h in XF DMEM medium (pH 7.4) supplemented with 1 mM pyruvate, 2 mM glutamine, and 10 mM glucose without CO_2_. Cells were then injected with 1, 10, or 20 μM of BAM15 or vehicle and the rates of oxygen consumption (OCR) and extracellular acidification (ECAR) were measured over 12 h. Data were normalized to nuclear double-stranded DNA content by staining live cells after assay with 20 μM Hoechst 33342 (ThermoFisher Scientific, Waltham, MA, USA) and reading fluorescence on an automated microplate reader (Biotek) at excitation/emission 350/461 nm. Maximal OCR was defined as the greatest rate achieved over the 12-h period. Time to maximal OCR was determined by subtracting the time of maximal OCR from baseline injection. The respiratory half-life (t_1/2_) was defined as the time by which the OCR had decayed to 50% of the maximal observed respiration as described previously [18].

#### Assessment of mitochondrial respiration in living cells

Mitochondrial function was determined in intact cells by real-time respirometry (Seahorse XFe24; Agilent). MDA-MB-231 and EO771 cells were seeded at 1.0 × 10^4^ cells/well in an XFe 24-well plate (Agilent). Once cells reached 80% confluency, they were treated with a vehicle (0.01% DMSO) or varying concentrations of BAM15 for 16 h. Following treatment, media was removed, and cells were incubated at 37°C for 1 h in XF DMEM medium (pH 7.4) supplemented with 1 mM pyruvate and 2 mM glutamine without CO_2_. Cells were then serially injected with 10 mM glucose, 1 μM oligomycin, 1 μM FCCP, and 0.5 μM rotenone and antimycin A. Components of mitochondrial function were calculated as described previously [67]. Data were normalized to nuclear content by staining live cells after assay with 20 μM Hoechst 33342 (ThermoFisher Scientific) and reading fluorescence on an automated microplate reader (Biotek) at excitation/emission 350/461 nm. Mitochondrial respiration was quantified as described previously [18].

#### Assessment of OXPHOS and ET capacity in permeabilized cells

OXPHOS and ET capacity were determined from MDA-MB-231 and EO771 cells as described previously [18, 68]. Briefly, cells were plated in 10-cm dishes, grown to 80% confluence. Once confluent, cells were treated with a vehicle (0.01% DMSO) or 20 μM BAM15 in differentiation medium for 16 h. After treatment, cells were suspended in MiR05 medium (Mitochondria respiration medium: 110 mM sucrose, 60 mM potassium lactobionate, 0.5 mM EGTA, 3 mM, MgCl2·6H2O, 20 mM taurine, 10 mM KH2PO4, 20 mM HEPES, and 2 mg/ml BSA, pH=7.1) [69]. A 2-mL suspension containing 1 million cells/mL was added into each chamber of an O2K high-resolution respirometer (Oroboros Instruments, Innsbruck, Austria). OXPHOS and ET capacity were measured in digitonin-permeabilized cells (10 μg/10^6^ cells using the following concentrations of substrates, uncouplers, and inhibitors: malate (2 mM), pyruvate (2.5 mM), ADP (2.5 mM), glutamate (10 mM), succinate (10 mM), tetramethyl-p-phenylenediamine (TMPD, 0.5 μM), ascorbate (2 mM), carbonylcyanide-p-trifluoromethoxyphenylhydrazone (FCCP, 0.5 μM increment), rotenone (75 nM), antimycin A (125 nM), and sodium azide (200 mM). OXPHOS and ET capacity were quantified as described previously [18, 68].

#### Substrate coupling control in permeabilized cells

S- and N-linked coupling control and respiratory capacity was determined in MDA-MB-231 and E0771 cells by high resolution respirometry. Cells were plated in 10-cm dishes, grown to confluence. Upon confluency cells were dissociated by trypsinization, transferred into conical tubes containing Hanks balanced salt solution (HBSS), and centrifuged at 350×*g* for 5 min at 25°C. The cell pellet was then resuspended in MiR05 medium. A 2-mL suspension containing 1 million cells/mL was added into each chamber of an O2K system (Oroboros Instruments). Oxygen flow was measured in digitonin-permeabilized cells in the presence of 2 mM pyruvate, 2 mM malate, and 10 mM glutamate or 10 mM succinate and 2.5 mM ADP followed by titration of BAM15 in 0.5 μM increments in the presence of 2.5 μM oligomycin. The acceptor and uncoupling control ratios were defined as OXPHOS (P)/LEAK (L) and ET (E)/ROUTINE (R), respectively. The ET/P ratio was calculated by dividing the rate of maximal uncoupled respiration by OXPHOS.

#### Superoxide Production

Superoxide production was determined in MDA-MB-231 and EO771 cells by addition of 100 mM of spin trap 5,5-dimethyl-1-pyrroline-N-oxide (Enzo Life Sciences) into the cell culture media for the duration of treatment [70]. The resulting conditioned media and crude cell extract were homogenized by sonication on ice and stored at −20°C until time of assay. Superoxide radicals were detected by electron paramagnetic resonance (Bruker EMX Plus spectroscope) in a quartz flat cell at room temperature. Instrument parameters were as follows: 20 mW microwave power, 1.0 G modulation amplitude, 1 × 10^5^ gain, 0.163-s time constant, and 80 G scan range. To improve signal-to-noise ratio, spectra were accumulated 4× for each sample. Quantitation was carried out by measuring and comparing the amplitudes of the first peaks on each spectrum.

#### Mitochondrial membrane potential (ΔΨm)

ΔΨm was determined in MDA-MB-231 and EO771 cells via flow cytometry using the fluorophore tetramethylrhodamine (TMRM) as described previously [19]. Briefly, cells were plated in 6-well dishes and grown to confluence. Once confluent, cells were removed by gentle scraping and transferred in 1xPBS into Eppendorf tubes, and centrifuged at 500×*g* for 5 min at 25°C. Cell pellets were suspended in TMRM solution (100 nM in PBS) and incubated at 37°C for 15 min protected from light. Cells were then centrifuged at 500×*g* for 5 min at 25°C. The cell pellets were suspended in BAM15, FCCP, or 0.01% DMSO vehicle solutions (in raw high glucose DMEM) and incubated at room temperature for 10 min protected from light. After incubation, cells were centrifuged at 500×*g* for 5 min at 25°C. Cell pellets were suspended in Annexin V solution (100 uL of 1x Annexin buffer and 5 uL of Annexin V per sample) and incubated for 10 min protected from light. TMRM geometric mean fluorescence intensity (GMFI) was assessed on a BD Accuri C6 flow cytometer equipped with a blue laser emitting light at a fixed wavelength of 488 nm and a red laser emitting light at a fixed wavelength of 640 nm. At least 100,000 cells were identified and electronically gated using the forward and side light-scatter mode, with Annexin V+ events excluded. Following acquisition, FCS files were transferred to a third-party software program (FCS Express v7.0, De Novo, Los Angeles, CA, USA) for analysis. Changes in mitochondrial membrane potential were assessed as a percentage change in TMRM GMFI between the vehicle control and the different treatments.

#### Tumor implantation

At 10 weeks of age, mice were lightly anesthetized with inhaled isoflurane (3–5% induction and 1–3% maintenance, shaved between the right 4th and 5th inguinal mammary glands, and orthotopically injected with E0771 cells (1 × 10^6^ cells in 60 μL of 1:1:1 Matrigel/Collagen I/PBS). Tumor growth was monitored twice weekly for the duration of the study by an electronic caliper and quantified by applying the formula [volume = 1/2 (length × width^2^); where length>width] for approximating the volume of an ellipsoid.

#### Animal study 1

At 12 weeks of age, lean mice bearing tumors ~100 mm^3^ were randomized to either a free-living or metabolic cage cohort. In each cohort, mice were randomized 1:1 by a blinded biostatistician according to body weight and tumor volume to 2.5 weeks of CTRL (standard diet) or BAM15 (BAM15; 0.1% w/w BAM15 suspended in a balanced diet). Daily food intake, body weight, and twice-weekly tumor dimensions were measured during treatment. At study end, mice were euthanized in their home cage by CO_2_ inhalation followed by cervical dislocation. Organ weights and size were measured at necropsy.

#### Animal study 2

At 12 weeks of age, mice with diet-induced obesity bearing tumors ~100 mm^3^ were randomized 1:1:1 by a blinded biostatistician according to body weight and tumor volume to 2.5 weeks of CTRL (HFD), BAM15 (BAM15; 0.1% w/w BAM15 suspended in HFD), or calorie restriction (CR; restriction of HFD via reduction in food intake). Daily food intake, body weight, and temperature and twice-weekly tumor dimensions were measured during treatment. At study end, mice were euthanized in their home cage by CO_2_ inhalation followed by cervical dislocation. Organ weights and size were measured at necropsy.

#### Oral gavage study

At 12 weeks of age, drug-naïve mice were randomized 1:1 to oral gavage of a vehicle (0.7% w/v methylcellulose, 2% v/v Tween-80, and 5% v/v DMSO) or 50 mg/kg BAM15 (50 mg/kg BAM15 in 0.7% w/v methylcellulose, 2% v/v Tween-80, and 5% v/v DMSO). A straight 22G gavage needle was introduced through the esophagus and the gavage administered as a bolus. The needle was then removed, and mice returned to their respective home cage. After 1 h, mice were euthanized in their home cage by CO_2_ inhalation followed by cervical dislocation. Tumors were then harvested and assayed by high-resolution respirometry.

#### Metabolic chamber experiment

Whole-body energy expenditure, oxygen consumption, carbon dioxide production, body weight, and physical activity were continuously monitored for the last 10 days period of treatment in a mouse metabolic chamber (Sable Systems) as described previously [18]. Briefly, animals were acclimated to the chamber for 7 days prior to data collection by placement into training cages identical to the metabolic chamber cage. Ad libitum access to food and water was continued while in the training and chamber cages. Average daily and cumulative data were calculated over the final 7 days in the chamber. Locomotor activity was determined by calculating the sum of all detectable motion (> 1 cm/s along *X*-, *Y*-, or *Z*-axis) over the continuous monitoring period.

#### Food intake

Food intake was measured to the nearest 0.1 g daily between 0900 and 1100 throughout the duration of the study as described previously [71]. Briefly, daily intake was calculated by subtracting the amount of diet recovered from the hopper weight, corrected for spillage found under the grid floor. Daily food intake per mouse was then averaged over treatment duration for CTRL and BAM15 and over the last 2 weeks of treatment for CR (to account for the food restriction titration over the first 4 days of treatment).

#### Body temperature

Body temperature was recorded via infrared thermometry during weighing (Fluke 572-2 Infrared Thermometer; Fluke, Everett, WA, USA) as described previously [18].

#### Body composition

Body composition was assessed before tumor implantation, at randomization and 2.5 weeks after treatment via nuclear magnetic resonance using LF110 BCA-Analyzer (Bruker Corporation, Billerica, MA, USA) as described previously [72]. Briefly, at ~0700, animals were removed from their cage and weighed. Animals were then placed in a restrainer and inserted into the NMR for approximately 2 min. Fat and fat-free mass were determined by calibration with internal standards according to manufacturer’s instructions.

#### Quantitative determination of BAM15 in mouse tissue

Frozen tissues were powdered in a tissue pulveriser (Cellcrusher, USA) cooled by liquid nitrogen. Powdered tissue samples were homogenized in 90% (v/v) acetonitrile (Sigma-Aldrich, 34851, Australia) and 10% (v/v) methanol (Sigma-Aldrich, Australia) using a motorized pellet pestle homogeniser (Sigma-Aldrich, Australia). Homogenate was centrifuged (800×*g* for 10 min) and supernatant collected. Serum was collected from whole blood by centrifugation. BAM15 was extracted by adding tissue homogenate supernatant/serum (1:9) to a solution of 90% (v/v) acetonitrile and 10% (v/v) methanol. The solution was briefly vortexed then centrifuged (18,000×*g* for 10 min). Supernatant was collected in auto-sampler vials (Thermo Fisher Scientific, Australia) for mass spectrometry. Standards were prepared by spiking known concentrations (0.1, 1, 10, and 100 ng) of BAM15 into untreated tissue or plasma samples prior to extraction. Liquid chromatography tandem mass spectrometry was performed on a Shimadzu Prominence LCMS-8030 (Shimazdu, Japan). Chromatographic separation was achieved using an ACUITY UPLC BEH, C18 column (Waters, USA). Mobile phase A consisted of 0.1% v/v formic acid (Sigma-Aldrich, Australia) in HLPC-grade water. Mobile phase B consisted of 0.1% v/v formic acid in acetonitrile. The analyte was eluted with a gradient of 5–80% mobile phase B at a flow rate of 0.4 mL/min with 10 μL injection volume electro-sprayed into the mass spectrometer. ESI was performed in positive mode. Primary transition of *m/z* 341 > 162 and secondary transition of *m/z* 341 >137 with 6-min retention time were used to identify BAM15. Quantification was determined by measuring peak areas using LabSolutions Software (Shimazdu, Japan) on the instrument. Concentrations of test samples were interpolated from a standard curve derived from the intensity values of standards.

#### Hematoxylin and Eosin, Ki-67, and TUNEL staining

Tumor sections were collected at necropsy. Tissues were grossed to size and fixed in 10% neutral buffered formalin for 72 h, changing the fixative every 24 h. Tissues were then paraffin embedded, sectioned to a width of 4 μm, and fixed to a glass slide. Slides were then stained for hematoxylin and eosin [73], Ki-67, or TUNEL positive and determined by immunohistochemistry. All tumor sections were evaluated and analyzed by a blinded pathologist.

For the Ki67+ staining, tumor sections were dewaxed, hydrated, and incubated in a heat-induced epitope retrieval (HIER) solution (pH 9.0 Tris-EDTA) for 20 min at 100°C. After cooling, tissue sections were incubated at 4°C overnight in anti-Ki-67 (IHC-00375) diluted 1:1000 in Leica BOND primary antibody diluent. Tissue sections were then washed, incubated at room temperature for 15 min in bond polymer, rewashed, and counterstained with 3,3′-Diaminobenzidine. Dehydrated tissue sections were imaged and quantified by a blinded investigator who manually counted the positive and negative cells to quantify the Ki67 index (Ki67+/Ki67- x100) in 4 randomly selected fields [74].

TUNEL (ab206386, Abcam) staining was performed per manufacturer protocol. Neoplasms were scanned at low magnification for areas of highest TUNEL labeling in non-necrotic areas. Five, 400× fields were examined within a 1-mm^2^ optical grid reticle (which encompasses approximately 200 cells) and a manual cell counter. TUNEL-labeled neoplastic cells within the grid were counted and averaged to quantify the TUNEL index.

#### Assessment of OXPHOS and ET capacity in tumor homogenates

OXPHOS and ET capacity were determined in ex vivo tumor homogenates by high resolution respirometry (Oxygraph-2k) as described previously [75]. During necropsy, a tumor biopsy (60 mg) was collected and immediately placed into ice cold BIOPS (50 mM K+-MES, 20 mM taurine, 0.5 mM dithiothreitol, 6.56 mM MgCl2, 5.77 mM ATP, 15 mM phosphocreatine, 20 mM imidazole, pH 7.1, adjusted with 5 N KOH at 0°C, 10 mM Ca–EGTA buffer, 2.77 mM CaK2EGTA + 7.23 mM K2EGTA; 0.1 mM free calcium) solution [69]. Tumors were cleaned by removing connective tissue, necrotic tissue, and adjacent fat from the tumor mass and cut into two small (~25mg) pieces. Tumor tissue sections were blotted dry on filter paper and weighed with a tared balance. Tissue sections were then placed into an ice-cold Dounce homogenizer containing 1 mL MiR05. The tissue was gently disrupted by completing 5 strokes with constant forward and reverse rotation until the homogenate appeared cloudy with little to no solid tissue remnants. The homogenate was transferred and brought up to 5-mL final volume with fresh, ice-cold MiR05. Remaining connective tissue was removed and subtracted from the wet weight. 2.25 mL of homogenate was added to each Oxygraph chamber, oxygen was injected into the chamber (~600 uM), and the flux was allowed to stabilize. OXPHOS and ET capacity were measured using the following concentrations of substrates, uncouplers, and inhibitors: malate (2 mM), pyruvate (2.5 mM), ADP (2.5 mM), glutamate (10 mM), succinate (10 mM), palmitoylcarnitine (10 μM), duroquinol (0.5 mM), tetramethyl-p-phenylenediamine (TMPD, 0.5 μM), ascorbate (2 mM), carbonylcyanide-p-trifluoromethoxyphenylhydrazone (FCCP, 0.5 μM increment), rotenone (75 nM), antimycin A (125 nM), and sodium azide ( 200 mM). OXPHOS and ET capacity were quantified as described previously [18, 68]. For the tumor gavage study, leak and electron transfer capacity were determined in the presence of malate (2 mM), pyruvate (2.5 mM), glutamate (10 mM), succinate (10 mM), and oligomycin (5 nM).

#### Citrate synthase activity

Citrate synthase activity was determined in tumor tissue using a commercially available colorimetric assay (Sigma-Aldrich, St. Louis, MO, USA) according to the manufacturer’s instructions. Briefly, frozen tumors (10 mg) were homogenized in 100 uL of ice cold 1X assay buffer using 10 strokes of a handheld homogenizer and incubated on ice for 10 min. Homogenized tumors were centrifuged at 10,000×*g* for 5 min at 4°C to pellet tissue debris. The supernatant was then transferred to a fresh tube, and protein content was assessed by BCA assay (Thermo Scientific). Twenty micrograms of protein lysate suspended in 1X assay buffer containing 30 mM acetyl CoA and 10 mM DTNB was plated in duplicate on a 96-well plate. Absorbance was then measured on a plate reader set to kinetic mode (412 nm, 1.5 min duration, 10-s intervals) before and after the addition of 10 mM oxaloacetate.

#### ATP content

ATP tissue concentration was determined in deproteinated tumor tissue using a commercially available fluorometric assay (Abcam) per the manufacturer’s instruction. Briefly, frozen tumors (10 mg) were homogenized in 100 uL ice cold 2N perchloric acid using 10 strokes of a handheld homogenizer and then incubated on ice for 45 min. The homogenized tumors were centrifuged at 13,000×*g* for 2 min at 4°C. The supernatant was then transferred to a fresh tube and the volume was brought to 500 uL with the ATP assay buffer. Excess PCA was precipitated by adding 100 uL of ice-cold 2M KOH, vortexing briefly, and maintaining a neutral pH. The samples were centrifuged at 13,000×*g* for 15 min at 4°C and the supernatant was collected for ATP measurement. Standards and samples were plated in duplicate into a 96-well black walled plate, the ATP reaction mix was added, and the plate was incubated at room temperature for 30 min protected from light. The reactions were analyzed with a microplate reader (Ex/Em = 535/587 nm). The ATP generating capacity of the OXPHOS system was derived by multiplying ATP content by the NS-linked OXPHOS rate.

#### Hydrogen peroxide activity

Hydrogen peroxide (H_2_O_2_) activity was determined in tumor homogenates by commercially available fluorometric assay (Cell Biolabs Inc., San Diego, CA) according to the manufacturer’s instructions. Briefly, tumor tissue (50 mg) was suspended in PBS and mechanically homogenized (Fastprep; MP Biomedicals). The resulting homogenates were spun at 10,000×*g* for 5 min to remove insoluble particles. The purified supernatant was decanted and diluted 3-fold in PBS. Diluted samples (50 μL) were incubated for 5 min in duplicate with catalyst reagent and 100 μL of the ROS probe dichloro-dihydrofluorescein DiOxyQ (DCFH-DiOxyQ) solution for an additional 30 min. The generated fluorescent product dichlorofluorescein (DCF) was measured on a Cytation 5 Cell Imaging Multi-Mode Reader (Biotek) at 480/530 nm excitation/emission wavelength. H_2_O_2_ concentrations were determined by fitting standards against a four-parameter logistic curve.

#### Quantification and statistical analysis

Data are reported as mean ± standard error of the mean (SEM) unless otherwise denoted in the figure legend. Statistical analysis was performed with Prism 8 (GraphPad, San Diego). Statistical procedures from individual experiments are detailed in the respective figure legends. Normality was assessed by Kolmogorov-Smirnov test. Significance was accepted as *P*<0.05.

## Supplementary Information

**Additional file 1: Table S1.** Related to Figure S2. List of gene symbols, adjusted p-values, and log2 fold changes after 16-hr treatment with BAM15. **Figure S1.** Related to Figure 1. BAM15-mediated mitochondrial uncoupling reduces cell viability, proliferation, and migration in human TNBC and murine luminal B breast cancer cells. **Figure S2.** Related to Figure 2. BAM15 reduces the expression of genes required for cellular proliferation and energy production in MDA-MB-231 cells. **Figure S3.** Related to Figure 3. BAM15 reduces OXPHOS and glycolytic capacity via ΔΨm destabilization in MDA-MB-231 and EO771 cells. **Figure S4.** Related to Figure 4. BAM15 suppresses tumor growth in C57BL/6J mice.

## Data Availability

The RNA sequencing datasets produced in this study are available at Gene Expression Omnibus (GEO) under the accession number GSE161502. Further information and requests for resources and reagents should be directed to and will be fulfilled by the Lead Contact, John P. Kirwan (john.kirwan@pbrc.edu).
