# Supplementary material for: Breast cancer growth and proliferation is suppressed by the mitochondrial targeted furazano[3,4-b]pyrazine BAM15
Source: Cancer Metab. 2021 Oct 9;9:36. doi: 10.1186/s40170-021-00274-5 (PMC8502397; doi:10.1186/s40170-021-00274-5)
Supplement: Supplementary file 1 — Additional file 1: Table S1. Related to Figure S2. List of gene symbols, adjusted p-values, and log2 fold changes after 16-hr treatment with BAM15. Figure S1. Related to Figure 1. BAM15-mediated mitochondrial uncoupling reduces cell viability, proliferation, and migration in human TNBC and murine luminal B breast cancer cells. Figure S2. Related to Figure 2. BAM15 reduces the expression of genes required for cellular proliferation and energy production in MDA-MB-231 cells. Figure S3. Related to Figure 3. BAM15 reduces OXPHOS and glycolytic capacity via ΔΨm destabilization in MDA-MB-231 and EO771 cells. Figure S4. Related to Figure 4. BAM15 suppresses tumor growth in C57BL/6J mice. [file 40170_2021_274_MOESM1_ESM.pdf]

Supplementary Tables and Figures

Breast Cancer Growth and Proliferation is Suppressed by the Mitochondrial Targeted Furazano[3,4-b]Pyrazine BAM15

Elizabeth R.M. Zunica, Christopher L. Axelrod, Eunhan Cho, Guillaume Spielmann, Gangarao Davuluri, Stephanie J. Alexopoulos, Martina Beretta, Kyle L. Hoehn, Wagner S. Dantas, Krisztian Stadler, William T. King, Kathryn Pergola, Brian A. Irving, Ingeborg M. Langohr, Shengping Yang, Charles L. Hoppel, L. Anne Gilmore, and John P. Kirwan

Table of Contents

Table S1. Related to Figure S2. List of gene symbols, adjusted p-values, and $\log_2$ fold changes after 16-hr treatment with BAM15.

Figure S1. Related to Figure 1. BAM15-mediated mitochondrial uncoupling reduces cell viability, proliferation, and migration in human TNBC and murine luminal B breast cancer cells

Figure S2. Related to Figure 2. BAM15 reduces the expression of genes required for cellular proliferation and energy production in MDA-MB-231 cells.

Figure S3. Related to Figure 3. BAM15 reduces OXPHOS and glycolytic capacity via $\Delta\Psi m$ destabilization in MDA-MB-231 and EO771 cells.

Figure S4. Related to Figure 4. BAM15 suppresses tumor growth in C57BL/6J mice.

Table S1. Related to Figure S2. List of gene symbols, adjusted p-values, and log_2 fold changes after 16-hr treatment with BAM15 relative to Vehicle.

Gene Symbol	Adjusted p-value	log2 Fold Change
Mitochondrial Dynamics		
DNM1L	0.004469624	-0.192639641
OPA1	0.000385584	-0.245923853
MFN1	0.142853387	-0.136827281
MFN2	0.075618866	-0.114323521
OMA1	0.044313266	0.289409181
PARL	7.44E-10	0.397058028
FIS1	0.094792673	-0.119268304
MFF	0.041530668	0.148740645
Autophagy		
SIRT1	7.77E-11	0.589486577
SIRT3	0.008517238	0.346885233
ULK1	3.32E-38	1.292392841
ATG2A	8.04E-16	0.579748321
ATG4B	3.36E-15	0.58085241
ATG16L2	0.333652842	0.133910611
ATG101	2.81E-22	0.773242444
Respiratory Complexes		
NDUFS1	5.52E-09	-0.402430818
SDHB	4.55E-09	-0.406957019
UQCRC2	0.000738109	-0.193959817
MT-CO1	0	2.775819976
ATP5F1A	5.62E-36	-0.593952869
Energy Metabolism		
PRKAA2	0.336169972	-0.091854631
PRKAB1	0.003175631	0.382836256
PRKAB2	2.13E-07	0.580965956
PRKAG1	1.39E-05	0.344592386
MTOR	0.800359432	0.023992445
AKT1	0.007062038	-0.265064537
AKT2	0.678432442	-0.033365888
AKT3	0.532525074	-0.079041635
PPARGC1A	0.848318919	-0.082656614
Glycolysis-1		
HK2	8.74E-15	-0.589816983
GPI	2.29E-17	-0.412208849
PFKL	0.000942345	-0.276006646
PFKM	0.176763982	-0.169734187

ALDOC	1.44E-05	-1.243202952
PGAM1	3.49E-17	-1.569936957
ENO1	9.15E-51	-0.694921947
PCK2	2.10E-117	2.06459341
GOT1	1.98E-50	0.850071749
PC	8.29E-12	-0.792722384
PDHB	9.66E-34	-0.691615092
LDHA	9.24E-105	-1.087817541
G6PC3	0.0002437	0.277650755
PGAM1	3.49E-17	-1.569936957
PGM1	5.68E-15	-0.498604195
PKM	2.48E-25	-0.647091972
TPI1	3.84E-27	-0.456179379
TCA Cycle		
ACLY	8.26E-09	-0.297696133
ACO1	6.41E-17	-0.494645596
ACO2	0.000452932	-0.236135866
CS	6.39E-18	-0.552642584
DLAT	7.47E-16	-0.499357187
DLD	1.44E-12	-0.441578066
DLST	0.001962104	-0.480756259
FH	1.44E-16	-0.514283398
IDH2	3.13E-07	-0.562788087
MDH1	6.53E-11	-0.397197499
OGDH	4.87E-05	-0.266060207
PDHA1	0.149301355	-0.081322737
PDHB	9.66E-34	-0.691615092
SDHA	3.33E-30	-0.607927994
SDHC	0.572099336	-0.033210501
SDHD	0.00036399	-0.763434684
SUCLA2	1.88E-05	-0.583563951
Lipid Metabolism		
CPT1A	0.001233066	0.169193678
CPT1C	0.223694298	-0.472465576
CPT2	8.21E-06	0.471011571
SLC25A20	0.006614826	-0.463162813
ACSL1	0.079631075	0.174320568
SLC27A4	0.204660875	-0.208894321
Insulin Receptor Signaling		
IRS1	6.69E-09	-0.382218846
IRS2	2.85E-42	0.652535829

FOXO3	4.63E-21	0.76992705
PIK3CD	1.94E-16	0.673662835
PIK3CA	0.000438935	0.304428544
MAPK1	0.07832594	-0.127537688
IGF1R	0.531800228	0.092496093
PDPK1	0.00836727	-0.281291215
Pentose Phosphate Pathway		
G6PD	0.011768938	0.16812385
H6PD	3.77E-06	0.384480307
PGLS	2.11E-12	0.489667725
PRPS1	2.74E-19	-0.666371009
RBKS	4.88E-08	0.651035464
RPE	0.325527261	-0.104079953
RPIA	0.451423311	0.073016357
TALDO1	0.006300912	0.137007751
TKT	4.24E-07	-0.404155146

Figure S1. Related to Figure 1. BAM15-mediated mitochondrial uncoupling reduces cell viability, proliferation, and migration in human TNBC and murine luminal B breast cancer cells

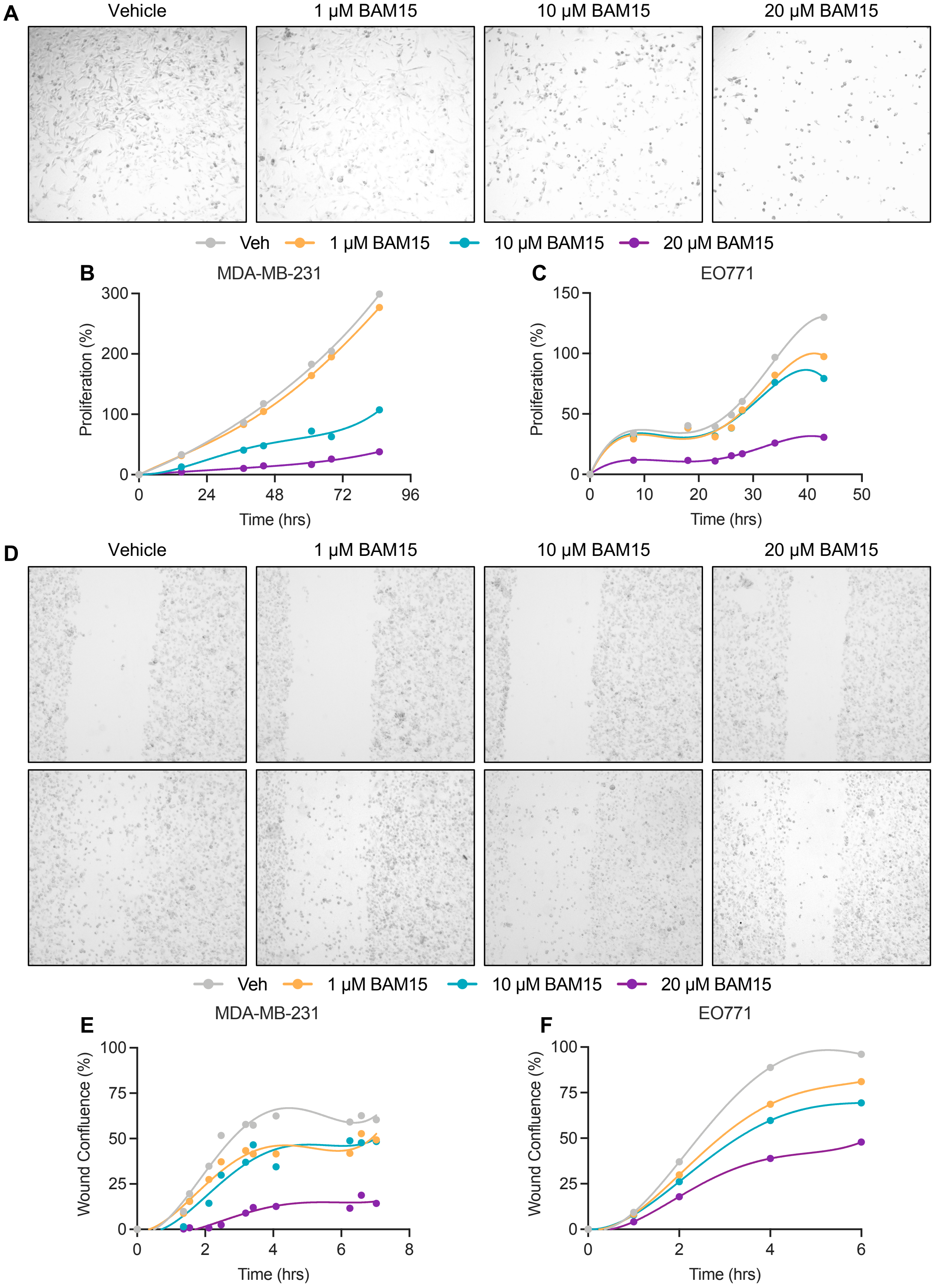

(**A**) Representative images of MDA-MB-231 cell proliferation after 91 hours of exposure to varying concentrations of BAM15. (**B**) Change in cellular proliferation over 4.5 days of continuous exposure to varying concentrations of BAM15 in MDA-MB-231 cells (N=10 for Veh, 2.5, 5, and 10 1 μM BAM15, N=11 for 0.5 and 1 μM BAM15, N=12 for 15 and 20 μM BAM15) and (**C**) two days in EO771 cells (N=10 per condition). (**D**) Representative images of MDA-MB-231 cell migration after 6 hours of exposure to varying concentrations of BAM15. (**E**) Wound confluence over 7 hours of continuous exposure to varying concentrations of BAM15 in MDA-MB-231 cells (N=3 per condition) and (**F**) EO771 cells. Panels B, C, E and F are shown as the mean fitted against a four-parameter logistic curve and were assessed by extra sum-of-squares F test.

Figure S2. Related to Figure 2. BAM15 reduces the expression of genes required for cellular proliferation and energy production in MDA-MB-231 cells.

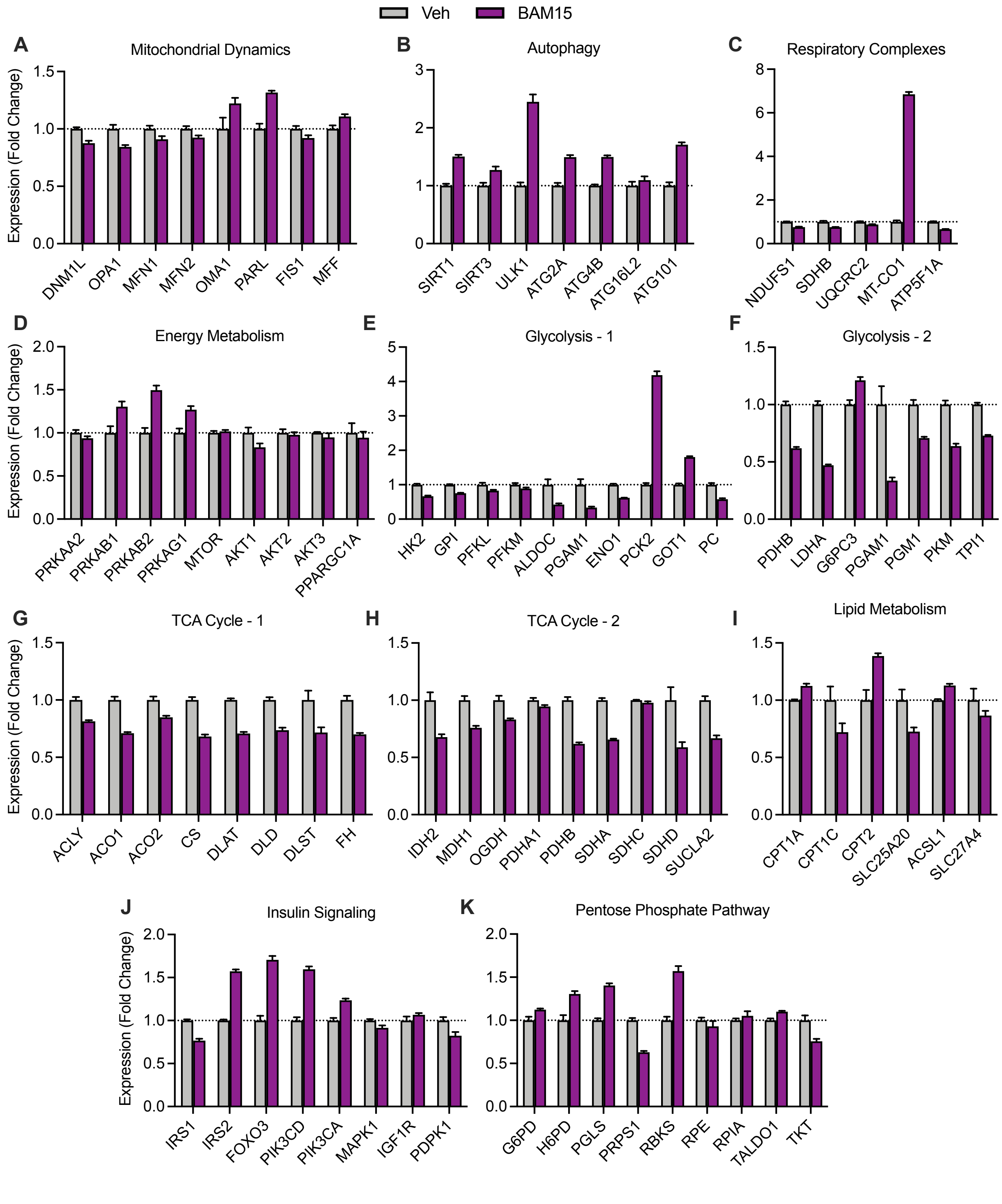

(A) Differential expression of genes related to mitochondrial dynamics, **(B)** autophagy, **(C)** respiratory complexes, **(D)** energy metabolism, **(E-F)** glycolysis, **(G-H)** TCA cycle, **(I)** lipid metabolism, **(J)** insulin receptor signaling, and **(K)** pentose phosphate pathway following 16-hr treatment with BAM15. Differentially regulated transcripts were filtered based upon the following criteria: $q<0.05$, base mean>30.

Figure S3. Related to Figure 3. BAM15 reduces OXPHOS and glycolytic capacity via ΔΨm destabilization in MDA-MB-231 and EO771 cells.

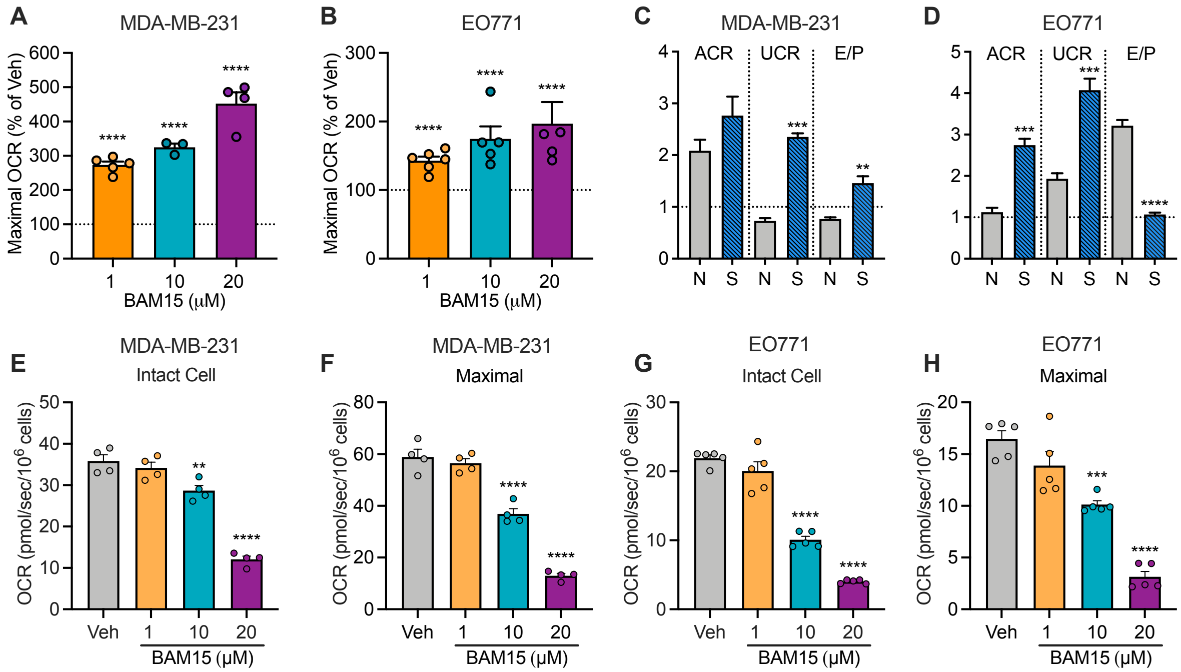

(**A-B**) Change in respiration (% of vehicle) following acute injection of varying concentrations of BAM15 in intact cells (1 μM BAM15 N=5, 10 μM N=3, and 20 μM N=4). (**C**) Acceptor control (**D**) uncoupling control, and (**E**) OXPHOS/ET ratios in digitonin-permeabilized cells (N=3 per treatment). (**E-H**) Intact and maximal cellular respiration following 16-hr exposure to Veh or varying concentrations of BAM15 as indicated in living cells (N=4-5 per condition as indicated). Data are shown as the mean ± SEM. *p<0.05, **p<0.01, ***p<0.01, ****p<0.001. Panels A, B, E, F, G, and H were assessed by one-way ANOVA with Tukey's multiple comparisons. Panels C and D were assessed by unpaired Student's t-test. Abbreviations: OCR, oxygen consumption rate; ECAR, extra-cellular acidification rate.

Figure S4. Related to Figure 4. BAM15 suppresses tumor growth in C57BL/6J mice.

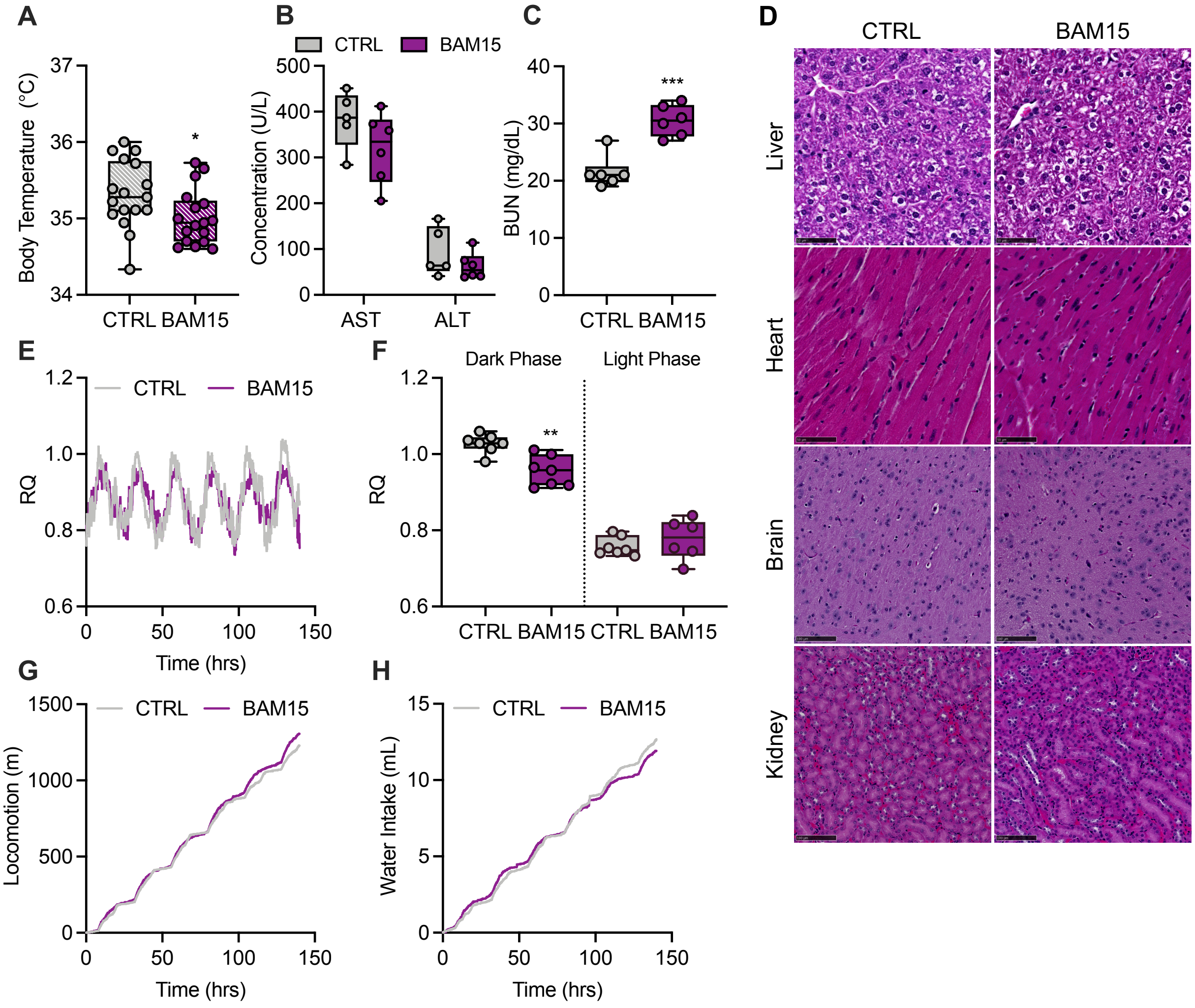

(A) Mean daily body temperature over the treatment period. **(B)** Plasma concentrations of AST and ALT, **(C)** BUN in CTRL and BAM15-treated animals (N indicated in individual figures). **(D)** Representative H&E of liver (scale = 50 μm), heart (scale = 50 μm), brain (scale = 100 μm), and kidney (scale = 100 μm) sections. **(E)** Change in the respiratory quotient (RQ) over a 6-day period and **(F)** mean RQ's during the dark and light phases (N=7 per group). **(G-H)** Change in locomotion and water intake over a 6-day period. Panels A, B, C, and F were assessed by unpaired Student's t-test. Abbreviations: BUN, blood urea nitrogen; RQ, respiratory quotient.
